# Multi-step ahead predictive model for blood glucose concentrations of type-1 diabetic patients

**DOI:** 10.1038/s41598-021-03341-5

**Published:** 2021-12-21

**Authors:** Syed Mohammed Arshad Zaidi, Varun Chandola, Muhanned Ibrahim, Bianca Romanski, Lucy D. Mastrandrea, Tarunraj Singh

**Affiliations:** 1 Computer Science and Engineering, University at Buffalo-SUNY, Buffalo, 14260 USA; 2 Mechanical and Aerospace Engineering, University at Buffalo-SUNY, Buffalo, 14260 USA; 3 Division of Pediatric Endocrinology, University at Buffalo-SUNY, Buffalo, 14203 USA; 4 Medical Information Technology, RWTH Aachen University, Pauwelsstr. 20, 52074 Aachen, Germany

**Keywords:** Type 1 diabetes, Biomedical engineering

## Abstract

Continuous monitoring of blood glucose (BG) levels is a key aspect of diabetes management. Patients with Type-1 diabetes (T1D) require an effective tool to monitor these levels in order to make appropriate decisions regarding insulin administration and food intake to keep BG levels in target range. Effectively and accurately predicting future BG levels at multi-time steps ahead benefits a patient with diabetes by helping them decrease the risks of extremes in BG including hypo- and hyperglycemia. In this study, we present a novel multi-component deep learning model BG-Predict that predicts the BG levels in a multi-step look ahead fashion. The model is evaluated both quantitatively and qualitatively on actual blood glucose data for 97 patients. For the prediction horizon (PH) of 30 mins, the average values for *root mean squared error* (RMSE), *mean absolute error* (MAE), *mean absolute percentage error* (MAPE), and *normalized mean squared error* (NRMSE) are $23.22 \pm 6.39$ mg/dL, 16.77 ± 4.87 mg/dL, $12.84 \pm 3.68$ and $0.08 \pm 0.01$ respectively. When Clarke and Parkes error grid analyses were performed comparing predicted BG with actual BG, the results showed average percentage of points in Zone A of $80.17 \pm 9.20$ and $84.81 \pm 6.11,$ respectively. We offer this tool as a mechanism to enhance the predictive capabilities of algorithms for patients with T1D.

## Introduction

Diabetes mellitus is a metabolic disease that causes the abnormal regulation of blood glucose (BG) levels in the body. Insulin, an endocrine hormone produced by the pancreas, facilitates uptake of glucose by a variety of cells. Type-1 diabetes (T1D) or insulin-dependent diabetes is an autoimmune condition requiring external administration of insulin for the regulation of blood glucose. Type-2 diabetes (T2D) results from insulin resistance or insulin insensitivity and the regulation of the BG levels require oral medications or insulin. Out of 463 million people with diabetes, approximately 1 million children and adolescents (under age 20) suffer from Type-1 diabetes (IAF diabetes atlas, 9th edition 2019, https://diabetesatlas.org/en/).

Although T1D cannot be cured or prevented, BG management is accomplished with subcutaneous administration of insulin either by injection or continuous infusion. Patients with T1D must monitor their BG level throughout the day and take necessary actions to prevent *hypoglycemia* (low BG levels) and *hyperglycemia* (high BG levels). The ability to accurately predict future BG levels would help patients prevent both low and high blood sugar levels and allow them to meet glycemic targets and decrease the risk of long-term complications. Factors that affect future BG levels include prior BG levels, insulin dose, meal intake, body mass index, physical activity among others. Incorporating all the features to predict BG levels with high accuracy poses a challenging task^1^. This is complicated by the physiological yet unpredictable variations seen in the BG levels caused throughout the day (dawn and dusk phenomena). For example, emotional stress can trigger hyperglycemia while physical activity can enhance insulin sensitivity thereby causing hypoglycemia for several hours. Capturing such variations in itself poses a unique challenge for any model to have enough predictive power to forecast BG levels.

Existing BG prediction approaches falls under two paradigms— *physiological-driven* and *data-driven*. Physiological-driven methods typically follow a compartment-based modeling approach, that requires extensive knowledge about the underlying mechanism for each individual^2^. The study^3^ provides a more comprehensive review on the use of physiological based approaches for modeling glucose-insulin systems. These approaches^4–6^ use insulin, meal intake, CGM signals and other variables such as physical activity, heart rate as inputs. Some studies^7,8^ used mathematical concepts such as fractional calculus for capturing complicated dynamics of BG measurements. Many of these models exploit the underlying processes of the system with the help of interconnected compartments that best explain the behavioural process between certain sub-system such as measuring glucose production and utilization, insulin action and meal absorption. These approaches also pose certain disadvantages such as identifying and establishing many parameters prior to making any predictions related to blood glucose values, making the model more cumbersome. Some of the ways to alleviate this issue is to make use of minimized version of these models^9,10^ or make use of machine learning techniques for identification of parameters^11^.

Data-driven approaches use an individual’s recorded historical data such as *Continuous Glucose Monitoring* (CGM) readings, meal intake information, programmed basal rate infusion amounts, etc., to learn a predictive model^12–14^. Data-driven approaches have the advantage of not having to model the underlying, and often unpredictable, physiological mechanisms. With tremendous advances in acquiring relevant medical data from sources such as CGMs^15^, insulin pumps^16^, and point of care (POC) devices^17^, data-driven approaches have shown promising results for BG prediction. With the deluge of information from different data sources, researchers from multidisciplinary fields have leveraged the use of different Machine Learning (ML) methods to identify relevant data points pertinent to predictive modeling in diabetes research.

In the past, statistical and machine learning (ML) techniques such as AutoRegressive Integrated Moving Average (ARIMA)^18^, Support Vector Regression (SVR)^19,20^, and Artificial Neural Networks (ANN)^21,22^ have been used for one-step ahead BG prediction. The existing prediction models using ML techniques can also be distinguished based on the different types of input data used. Some studies^23,24^ explored the use of random forests while^25^ used support vector regression for predicting future BG by using the historical BG along with meal intake and insulin concentrations. On the other hand, some studies^2,26^ investigated the use of ARIMA models such as fixed-order ARIMA^27^ and adaptive-order ARIMA^18,28^ for predicting future BG based only on the historical BG information. These models, however, perform poorly for long-term forecasting since they do not effectively capture long-term dependencies. Recently, deep recurrent neural network architectures, such as the *Long Short-Term Memory* (LSTM) networks, have received considerable attention since they handle long-term dependencies well and effectively outperform any of traditional machine learning approach for predicting blood glucose with prediction horizon of 30 mins^29–31^. Sun et al.^29^ used Long Short-Term memory (LSTM) based approach for predicting blood glucose levels for different prediction horizons. Mhasker et al.^30^ developed a semi-supervised deep convolutional network for effectively predicting BG with prediction horizon of 30 mins. This approach makes the use of a judge predictor based on function approximation on data defined manifolds, using diffusion polynomials. Daskalaki et al.^31^ proposed a real-time adaptive recurrent neural network-based model that uses BG and insulin information for predicting future BG values with prediction horizons of 30 mins and 45 mins. These studies highlight the emergence of advanced ML-based tools in the area of BG prediction in diabetes. Additionally, innovative approaches for forecasting BG are essential for the development of effective artificial pancreas and personalized diabetes support systems.

Though these recurrent neural networks, like LSTMs, have become the preferred tools for sequence modeling tasks, such as language translation^32^, they are shown to have some drawbacks, primarily in memorizing long temporal sequences. One of the drawback is related to memorizing a very long temporal sequence since a lot of information that passes between the cells of the network doesn’t propagate well. In addition to this, when a input is fed to each LSTM cell at every time-step, it consumes a lot of time during training. In order to address this issue, the use of 1D Convolutional Neural Network (CNN) has proven to be a better modeling approach for temporal sequences by coupling convolution operation and dilation rate of the filters. This provides a great advantage of capturing larger receptive field of the input sequence. In fact, recent studies have shown that using a one-dimensional Convolution Neural Network (CNN) can better model long-range temporal dependencies^33^. This has led to the use of Temporal Convolution Neural Networks (TCNs) over LSTMs for many time series prediction tasks that involve long-term memories^33^.

We present a novel TCN based predictive model that can effectively capture long-term history of an individual for BG prediction, and report significant improvements over state of art BG prediction models for a 30 mins prediction horizon. The salient aspects of the proposed model include: (a) multi-step prediction that better captures the temporal auto-correlations in the output compared to traditional one-step prediction schemes, (b) multi-compartment model that uses varying length histories for different inputs and their combinations, and, (c) individual-specific model training to learn the individual characteristics for BG prediction. To the best of our knowledge this is the first work focusing on modeling future blood glucose based on varying past history information on blood glucose, food intake, basal and bolus insulin using the proposed approach. The illustrated experimental results derived from clinical data for 97 T1D patients show the effectiveness of our proposed model in forecasting BG with prediction horizon of 30 mins.

## Results and discussion

The experiments were conducted on Tidepool (More information on https://www.tidepool.org/) datasets provided by the Juvenile Diabetes Research Foundation (JDRF) (More information on https://www.jdrf.org/). The datasets consists of real world data for 97 patients with T1D patients. For each patient, an unseen test data set was used for evaluating the performance of the model. We computed the following quantitative measures of error—*root-mean-square error (RMSE)*, *mean-absolute-error (MAE)*, *mean absolute percentage error (MAPE)* and *normalized root mean square error (NRMSE)*. We also computed these measures for three different categories within the data set. These three subsets pertain to actual blood glucose concentrations falling in different range values. The categories were as follows: (1) *Hypoglycemic* range with blood glucose less than 70 mg/dL, (2) *Normoglycemic* range with BG values greater than or equal to 70 mg/dL and less than 180 mg/dL, and (3) *Hyperglycemic* range with BG readings greater than or equal to 180 mg/dL.

This study investigates the impact and use of deep learning approaches for modeling blood glucose levels using a multi-step approach. This approach will provide well-informed analysis as well as support to individuals with Type 1 diabetes. For the prediction horizon (PH) of 30 mins, the proposed model has been evaluated per patient both quantitatively and qualitatively with respect to different performance measures described in the evaluation metrics section.

The detailed quantitative results for 30 mins ahead forecasting can be found as Supplementary Table S6. The table provides a summary of both the overall and detailed quantitative performance of the model to predict BG levels 30 mins in the future. In the table, some of the patients had no hypo- or hyperglycemic test samples and this has been indicated as *No test samples*. Quantitatively, the proposed method shows the overall average value to be $23.22 \pm 6.39$ mg/dL in root mean squared error (RMSE), $16.77 \pm 4.87$ mg/dL in mean absolute error (MAE), 12.84 ± 3.68 in mean absolute percentage error (MAPE) and $0.08 \pm 0.01$ in normalized mean squared error (NRMSE). For Hypoglycemia, the average value is $21.65 \pm 10.42$ mg/dL in root mean squared error (RMSE), $15.62 \pm 8.39$ mg/dL in mean absolute error (MAE), 26.13 ± 14.08 in mean absolute percentage error (MAPE) and 0.07 ± 0.03 in normalized mean squared error (NRMSE). For Hyperglycemia, the average value is $30.47 \pm 9.35$ mg/dL in root mean squared error (RMSE), $22.84 \pm 7.66$ mg/dL in mean absolute error (MAE), $10.55 \pm 3.77$ mg/dL in mean absolute percentage error (MAPE) and $0.10 \pm 0.05$ in normalized mean squared error (NRMSE). For Normoglycemia, the average value is $21.80 \pm 5.90$ mg/dL in root mean squared error (RMSE), $15.90 \pm 4.71$ mg/dL in mean absolute error (MAE), $13.29 \pm 4.00$ in mean absolute percentage error (MAPE) and $0.07 \pm 0.01$ in normalized mean squared error (NRMSE).

In terms of accuracy, the overall true positive rate for hypoglycemia, hyperglycemia and normoglycemia is $0.48 \pm 0.23,$ $0.73 \pm 0.17$ and $0.88 \pm 0.08$ respectively. On the other hand, the overall false positive rate for hypoglycemia, hyperglycemia and normoglycemia is $0.05 \pm 0.05,$ $0.04 \pm 0.04$ and $0.26 \pm 0.11$ respectively. In general, the model was found to be difficult in capturing the hypoglycemic events while capturing the hyper- and normo-glycemic events well. The false positive rates for all the three events were pretty low indicating that the model didn’t raise a large number of false alarms while performing the blood glucose forecasting at 30 mins ahead. The qualitative results of clinical or zonal accuracy for all the patients in terms of Clarke and Parkes error grids can be found as Supplementary Figs. S1 and S2 respectively. In Clarke and Parkes error grid analysis, the average percentage of points lying in Zone A were $80.17 \pm 9.20$ and $84.81 \pm 6.11$ respectively. The model consistently provides accurate result as indicated by predicted BG values in Zone A in both the Clarke and Parkes error grid.

Overall, these findings indicate that the blood glucose can be effectively estimated for a 30 mins prediction horizon for individual patients. This model has an inherent advantage of taking multi-view of the data and we can also leverage other information such as physical activity that can further enhance the predictive accuracy of blood glucose levels at prediction horizon of 45 mins and 50 mins. Amongst the related approaches, Marcus et al.^34^ presented the use of kernel ridge regression (KRR) approach in predicting the future BG at 30 mins with just the past history of BG values for individual patient. The model was validated on 11 T1D patients and their reported average RMSE values is 20.48 mg/dL. Mhasker et al.^30^ make use of non-patient specific semi-supervised deep learning approach for predicting future blood glucose values. This study only included the subject age group children less than 18 years old and didn’t include the prediction of blood glucose measurements. Mirshekarian et al.^11^ used long short-term memory (LSTM) networks to predict the blood glucose for 10 T1D patients. The subject age group in this study wasn’t reported, however, the RMSE for 30 mins was reported to be 21.4 mg/dL.

**Figure 1:**
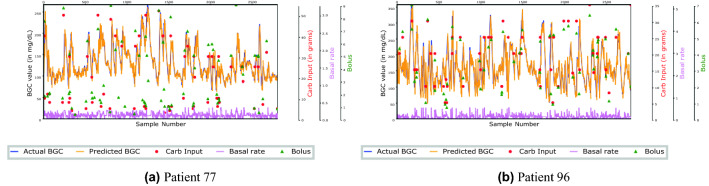
30 mins forecasting on validation data for two T1D patients.

Figure 1 shows the prediction accuracy of the model 30 mins ahead on validation data for two T1D patients. It can be seen that the model is able to capture the hypoglycemic and hyperglycemic events well.

### Comparison with other approaches

Table 1 presents the results of comparison of our model, BG-Predict with other baseline approaches in terms of RMSE, MAE, MAPE and NRMSE. The units for RMSE, MAE are in mg/dL.

1. **Naive Forecasting** In naive forecasting, the last recorded observation of blood glucose concentration is copied to the 30 mins ahead and the model is evaluated with the actual blood glucose concentration. This can be considered as a baseline method in comparing other methods.
2. **Gaussian Processes (GP)** Gaussian Process is a bayesian non-parametric approach that generalizes the Gaussian distribution of functions and can be used for both regression and classification. We use the Gaussian Process regression model for forecasting blood glucose concentration 30 mins ahead.
3. **Kernel Ridge Regression (KRR)** Kernel Ridge Regression (KRR) is a non-parametric model that combines ridge regression (linear least squares and $L_2$-norm regularization) with the kernel trick. The model learns the function in a space induced by the choice of kernel and the data. We used Linear and Radial Basis Function (RBF) kernel for forecasting blood glucose concentration 30 mins ahead.

The inputs to the KRR and GP models are 3 h history of basal, bolus and meal intake and 30 mins history of blood glucose measurements. For both KRR with linear and RBF kernels we did an exhaustive grid search over several hyperparameter values with gamma = [0.01, 0.1, 1.0, 10, 100.0] and alpha = [1.0, 0.1, 0.01, 0.001]. We found alpha = 1.0 and gamma = 0.1 to be the best choice of hyperparameter values. The kernel used for the GP regression model was Automatic relevance Determination (ARD) Radial Basis Function (RBF) with length scale and variance initialized to 1.0.

**Table 1:** Comparison with other approaches.

Model approach	RMSE	MAE	MAPE	NRMSE
Naive forecasting	$31.12 \pm 8.49$	$21.96 \pm 6.11$	$16.92 \pm 4.80$	$0.12 \pm 0.02$
GP	$28.01 \pm 7.39$	$20.05 \pm 5.39$	$15.09 \pm 3.88$	$0.11 \pm 0.02$
KRR (linear)	$25.77 \pm 6.62$	$18.75 \pm 4.90$	$14.31 \pm 3.65$	$0.10 \pm 0.01$
KRR (RBF)	$32.45 \pm 8.62$	$23.21 \pm 6.20$	$17.22 \pm 4.12$	$0.13 \pm 0.02$
BG-predict	$23.22 \pm 6.39$	$16.77 \pm 4.87$	$12.84 \pm 3.68$	$0.08 \pm 0.01$

We also compared our proposed model with AutoRegressive with exogenous variables (ARX) and AutoRegressive Moving Average with exogenous variables (ARMAX), which are competitive models for glucose prediction as indicated in Table 2. ARX and ARMAX models are time series models that is an extension of the AutoRegressive (AR) and AutoRegressive Moving Average (ARMA) models, which incorporates other exogenous variables. Specifically, it takes the history of an endogenous variable along with history of external exogenous variables to determine the future values of the endogenous variables. The inputs to the ARMAX models are 3 h (36 timesteps of 5 mins each) history of basal, bolus and meal intake and 30 mins (6 timesteps of 5 mins each) history of blood glucose measurements. We selected the polynomial orders, na = 6, nb = [36 36 36], nc = 6, nk = [0 0 0] for ARMAX model and na = 6, nb = [36 36 36], nk = [0 0 0] for ARX model. The parameter estimation was performed using nonlinear least squares with automatically chosen line search method incorporated in the system identification toolbox in Matlab. We observed the performance of ARMAX model to be better than ARX and our proposed model in terms of the quantitative evaluation metrics, however, when we compared the true positive rate or sensitivity under hypoglycemia and hyperglycemia, then our model performed much better than both the ARX and ARMAX model. This is an indication that the ARX and ARMAX models are performing well only under normoglycemia but is unable to capture the important events for the prediction horizon of 30 mins.

**Table 2:** Comparison of ARMAX model with the proposed model in terms of true positive rate (TPR) under hypoglycemia and hyperglycemia.

Model approach	RMSE	MAE	MAPE	NRMSE	TPR-hyper	TPR-hypo
ARX	$20.31 \pm 5.01$	$14.81 \pm 3.75$	$11.61 \pm 2.87$	$0.07 \pm 0.01$	$0.70 \pm 0.17$	$0.17 \pm 0.12$
ARMAX	$19.25 \pm 1.12$	$16.35 \pm 1.18$	$13.00 \pm 1.65$	$0.08 \pm 0.02$	$0.64 \pm 0.17$	$0.30 \pm 0.46$
BG-predict	$23.22 \pm 6.39$	$16.77 \pm 4.87$	$12.84 \pm 3.68$	$0.08 \pm 0.01$	$\mathbf {0.73 \pm 0.17}$	$\mathbf {0.48 \pm 0.23}$

We have used the two-sample t-test with unidentical variance for statistical testing and have reported the p-value for interpreting the comparison of the models corresponding to the quantitative evaluation measures. The results have been shown in Table 3. It can be seen that all the p-values are very small and much lower than the general significance threshold of 0.05 which implies that the results of the models are different.

**Table 3:** Statistical testing results showing p-values for making comparison on the results of different models in terms of evaluation measures.

Models		Evaluation measures			
Model 1	Model 2	RMSE	MAE	MAPE	NRMSE
BG-predict	Naive	1.21e−14	6.44e−13	3.72e−13	1.53e−39
BG-predict	GP	1.07e−11	1.70e−10	2.54e−09	2.13e−38
BG-predict	KRR (linear)	7.36e−05	7.32e−05	0.0001	4.51e−29
BG-predict	KRR (RBF)	9.68e−23	1.27e−21	1.00e−21	6.34e−62
BG-predict	ARX	5.00e−60	5.14e−58	7.19e−56	3.48e−55
BG-predict	ARMAX	2.48e−69	1.26e−50	1.15e−46	1.07e−37

### Comparison for different prediction horizons

Supplementary Table S3 presents the comparative evaluation of model’s results on prediction horizons of 30 mins and 60 mins for 24 patients (i.e. Patients ID 74–97). We selected 24 out of 97 patients to show the performance of the proposed model for prediction horizon of 30 and 60 mins on patients data that have above average quality. These patients were selected based on the below quality criteria: (i) Time in range > 70%—It is likely that meal information in datasets showing a high time in range (70–180 mg/dL) is more accurate, as determining the carbohydrate content of meals correctly is fundamental for good blood glucose control, (ii) Average number of meal inputs per day > 3.5—A high average number of daily meal inputs can only be observed in datasets with regular reported meals and (iii) Time in hypoglycemia < 2%—There seems to be cases in the data where carbohydrates which are consumed to counteract hypoglycemia are often not reported. Hence, it is likely that there are fewer missing meal labels in datasets showing a shorter time in hypoglycemia (< 70 mg/dL).

For prediction horizon of 30 mins, the model shows the average value of $19.18 \pm 3.29$ in root mean squared error (RMSE), 13.42 ± 2.36 in mean absolute error (MAE), $9.91 \pm 1.61$ in mean absolute percentage error (MAPE) and $0.07 \pm 0.01$ in normalized mean squared error (NRMSE). For prediction horizon of 60 mins, the model shows the average value of $32.06 \pm 5.81$ in root mean squared error (RMSE), $23.40 \pm 4.50$ in mean absolute error (MAE), $17.31 \pm 2.86$ in mean absolute percentage error (MAPE) and 0.12 ± 0.02 in normalized mean squared error (NRMSE). It is indicative from the table and these values that as we increase the prediction horizon, the model’s performance drops as we expected.

### Sensitivity analysis of features and past history

Supplementary Table S4 describes the impact of features and past history on the blood glucose output at prediction horizon of 30 mins. This describes the impact of simultaneous inclusion/exclusion and past history (p) of input variables of each component on the output. We did the analysis on some of data for patient 77 and we monitor the evaluation measure of RMSE.

For the first component, we noticed as we switched off just the meal intake component, the performance on the predictions is slightly decreased. Similarly, we also noticed a decrease in RMSE when we switch both the components, (no history or p = 0). There was no clear trend as we increase the past history of the variables in the first component, but there is a slight decrease and then a gradual increase as we move past the history of timesteps. For the second component, we noticed a slight decrease in evaluation measure as we switch off the only BG variable while when we switch on the variable, we notice a slightly decrease and increase in the performance of the model.

For the third component, when we switch off both the basal and the BG variables, then we observed a slight decrease in RMSE as compare to when we switched on both the components. For the fourth component, we noticed a decrease in the RMSE measure when we switch off both the BG and the bolus variables. When we switch off just the bolus variable then also we noticed a slight decrease in performance in comparison to when we switched on both the variables.

### Weighted contribution on the predictions

Supplementary Table S5 presents the contribution of weights of each component on the predicted output sequence. We provide the analysis for 24 patients (i.e. Patients ID 74–97). For forecasting of prediction horizon of 30 mins, we observed that the contribution of the first component (3 h history of meal intake and blood glucose) and the second component (recent 30 mins history of blood glucose) to be more in comparison to the third component (3 h history of basal insulin and blood glucose) and the fourth component (3 h history of bolus insulin and blood glucose).

## Methods

### Problem formulation

Given the historical data inputs of *meal intake* (M), *blood glucose* (BG), *basal insulin rate* (Ba) and *bolus insulin* (Bo), we want to predict the future BG values in multi-step manner. The problem can be formulated as:

- Given $$\{\text {M}_{0}, \dots , \text {M}_{t}\}$$; $$\{\text {BG}_{0}, \dots , \text {BG}_{t}\}$$; $$\{\text {Ba}_{0}, \dots , \text {Ba}_{t}\}$$ and $$\{\text {Bo}_{0}, \dots , \text {Bo}_{t}\}$$
- Predict $$\text {BG}_{t+1}, \dots , \text {BG}_{t+f}$$

We define our model by accounting the different input history length of these input variables. Considering the different input history, we reformulate the problem as:

- Given $$\{\text {(M, BG)}_{t - p_1}, \dots , \text {(M, BG)}_{t}\}$$; $$\{\text {BG}_{t - p_2}, \dots , \text {BG}_{t}\}$$; $$\{\text {(Ba, BG)}_{t - p_3}, \dots , \text {(Ba, BG)}_{t}\}$$ and $$\{\text {(Bo, BG)}_{t - p_4}, \dots , \text {(Bo, BG)}_{t}\}$$
- Predict $$\text {BG}_{t+1}, \dots , \text {BG}_{t+f}$$

We denote *f* as the prediction horizon (PH) while the starting position in history for the inputs sequence length in the four components of our proposed model is denoted by $p_1$, $p_2$, $p_3$ and $p_4$. Here, $(\cdot , \cdot )$ denotes the interaction term indicating the multiple variables for all time steps to be fed to a component of the proposed model. For example, $\text {(M, BG)}$ implies considering both input *meal intake* and *blood glucose* variables for all time steps for sequence length indexed from $t - p_1$ until *t*.

### Data

The experiments in our study are conducted on data for 300 patients with T1D. All methods were carried out in accordance with relevant guidelines and regulations. The experimental analysis was conducted on anonymized datasets obtained from the Tidepool Big Data Donation Project (More information on https://www.tidepool.org/bigdata). These datasets do not require IRB approval for use due to the HHS Policy for Protection of Human Research Subjects exemption research guideline, 45 CFR 46.104 (More information on https://www.hhs.gov/ohrp/regulations-and-policy/regulations/45-cfr-46/common-rule-subpart-a-46104/index.html).

**Figure 2:**
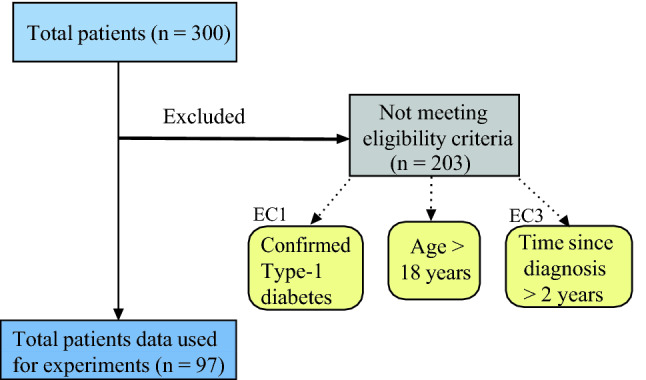
Flowchart for processing tidepool data.

Data of patients using hybrid closed loop systems is included. The training and the testing data for each patient were separately provided to us by Tidepool. Each dataset consists of information on CGM readings (sampling time 5 mins), basal and bolus insulin administration and carbohydrate intake. Some datasets contain additional information on CGM readings or performed physical activity (type, duration and/or distance travelled). Additional information including age, number of years living with diabetes, diabetes type, and biological sex is available for each patient. Since the purpose of this study is to model the blood glucose dynamics of adult patients with type-1 diabetes, we adopt a strategy as shown in Fig.  2 for identifying eligible patients for our experiments. The inclusion of the patients is made on the basis of some Eligibility Criteria (EC). The criterions are: (1) *EC1*: Patients with confirmed type-1 diabetes, (2) *EC2*: Patients of age > 18 years, and (3) *EC3*: Patients whose time since diagnosis > 2 years.

During the data preprocessing stage, there were some missing CGM readings. It is assumed that the time course of the blood glucose level within a small time window, i. e. up to 30 mins, can be extrapolated from surrounding blood glucose levels at a sufficient accuracy. To ensure equidistant data points, such short gaps are filled using piecewise cubic interpolation. Larger gaps are not filled by interpolation, as there is a risk of missing significant oscillations of the blood glucose level. Instead, they are used to split the dataset into subsets. Any subset containing a calibration, which differs significantly (> 50 mg/dL) from the simultaneous CGM reading, is dismissed, as the calibration likely causes a step discontinuity in CGM readings, which does not represent the actual time course of the blood glucose level.

The characteristics of the data for each patient can be found in Supplementary Tables S1 and S2. Each of the patient’s data is divided into training and testing dataset each of which consists of *CGM recordings*, *Basal*, *Normal Bolus*, *Meal Intake*. Supplementary Table S1 provides the number of CGM samples, mean CGM, frequency of meals taken, hypo-, hyper- and normo-glycemic values in training and test data for each patient. In each of the patient’s data, the observation value for CGM measurement, meal intake, basal and bolus insulin is recorded every 5 mins.

Supplementary Table S2 provides the duration (in days), standard deviation of CGM, Coefficient of variation of CGM, Time spent (in h) for hypo- hyper- and normo-glycemia. The average duration (in days) for the train and test data of the study population is $143.42 \pm 90.39$ days and $25.37 \pm 4.96$ days respectively. The CGM’s average and coefficient of variation for the train data of the study population is $139.05 \pm 26.17$ mg/dL and $0.31 \pm 0.06$ respectively. The CGM’s average and coefficient of variation for the test data of the entire population is $140.60 \pm 22.78$ mg/dL and $0.32 \pm 0.06$ respectively. The average time (in h) spent in hypoglycemia for the train and the test data of the study population is $112.69 \pm 156.53$ h and $19.88 \pm 18.90$ h respectively. The average time (in h) spent in hyperglycemia for the train and the test data of the study population is 666.95 ± 766.21 h and $115.25 \pm 83.70$ h respectively. The average time (in h) spent in normoglycemia for the train and the test data of the study population is $2664.48 \pm 1878.40$ h and $474.03 \pm 123.69$ h respectively.

### Temporal convolutional networks (TCN)

Sequence-based prediction tasks are solved using Recurrent neural networks (RNN) like (Long short-term memory) LSTM networks and Gated recurrent unit (GRU) networks in the deep learning world. Although these approaches were quite successful in solving several sequence-to-sequence tasks, we encountered certain limitations such as inability to handle long time sequences as well as vanishing and exploding gradients. Temporal convolutional networks (TCN) were introduced for video-action segmentation task^35^ to overcome the issues faced by the RNN-based approaches. TCN can be seen as the combination of 1D convolutional neural network with dilated and causal convolutions.

#### Causal and dilated convolutions

The primary characteristics of temporal convolutional network is that the convolutions are causal and dilated. Causal convolution implies that the convolution operation output at time *t* in a current layer is performed by considering elements at time *t* and earlier in the previous layer. Causal convolutions ensure that there is no information leakage from future to present while computing the output prediction at time *t*. By just performing the simple causal conventional convolution operation, we are only looking back at the history of sequence with size linear in the depth of the network. This makes the task more challenging for longer input sequences. In order to alleviate this problem, dilated convolutions were introduced to enable large receptive field in^33^.

Use of dilated convolutions implies applying the convolution filter to an area larger than its size by enhancing the filter by dilating it with zeros. Alternatively, the filter can be applied over the larger area in the input by skipping input values with a certain step or dilation factor. Formally, dilated convolution is defined as:

$$\begin{aligned} F(s) = ({\mathbf {x}} *_d f) = \sum _{i = 0}^{k - 1} f(i)\cdot {\mathbf {x}}_{s - d.i} \end{aligned}$$

**Figure 3:**
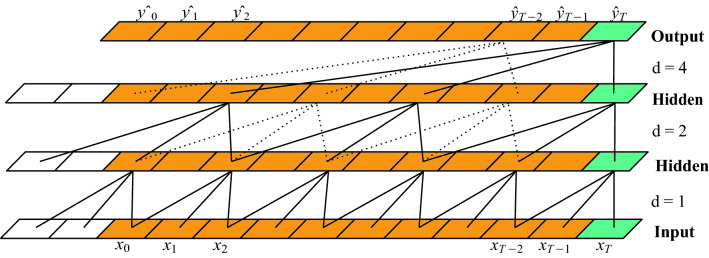
Dilated causal convolutions with dilation factor $d = 1, 2, 4$, and kernel size, $k = 3$.

Here, ${\mathbf {x}}$ is the input sequence, *d* is the dilation factor, *f* is the filter and *k* is the filter size. *F* is the dilated convolution operation, *s* is an element of the input sequence, ${\mathbf {x}}$, $s - d.i$ accounts for the direction of the past. The use of dilated convolution serves two purposes, *firstly* the output of each convolution propagates rich information while tracking very long data sequence. As the dilation factor is increased, the range of inputs is increased and have increased receptive field and *secondly*, it is efficient in computation as the computation cost is less as compare to the use of larger filter size to increase the receptive field. Dilated convolutions are shown in Fig.  3 with dilation factors *d* and kernel size *k*.

#### Residual network

Deep neural networks are popularly used in several studies related to image processing. As we stack more layers, there is great improvement in results, however, there are problems related to vanishing/exploding gradients^36,37^. Although, these problems can be alleviated with different solutions such as initialization strategies^38^, etc., there are still issues related to degradation, i.e. arising of higher training error, as we add more layers to the network. With the advent of residual block^39^, we can achieve more deep layered network without leading to degradation. Instead of stacking layers and learning the desired (true) mapping function ${\mathcal {H}}(x)$ like in the conventional neural network block, the layers of the residual block tries to learn the residual function ${\mathcal {F}}(x)$, i.e., ${\mathcal {F}}(x) = {\mathcal {H}}(x) - x$. The output of the residual block will lead to the learning of desired mapping function, which can be recast as ${\mathcal {H}}(x) = {\mathcal {F}}(x) + x$. This expression can be realized with the help of shortcut/skip connections. This allows to skip one or more layers without introducing any extra parameter thereby avoiding any further increment to computational complexity. Since, the use of deeper and larger temporal convolutional neural network helps in computation involving larger receptive fields, it would be important to make sure that this large and deep network remains stable throughout training. In order to do this, the use of residual block is kept in the TCN based model.

**Figure 4:**
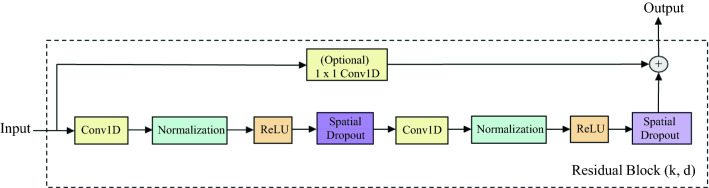
Residual block in temporal convolutional network.

Figure 4 shows the structure of the residual block that is incorporated in each of the hidden layer in the temporal convolutional network. The residual block in each of the hidden layer differs in terms of dilation factors *d*. The residual block consists of two 1D convolutional layer with same dilation factor *d* and kernel size *k* for better capturing long-term temporal dependencies. Each of which is followed by the normalization, activation and dropout layer. For the normalization block, we can use either the layer or batch normalization. Layer normalization^40^ is used to normalize the activations of the previous layer for each given example independently in a batch rather than across batch as seen in batch normalization^41^. The Relu block comprises of the ReLU (Rectified Linear Unit) activation, which is a non-linear and differentiable activation function that is useful for learning complex, non-linear mapping of data. We use a 1D version of spatial dropout^42^, a regularization technique like dropout but which differs in its working operation. In the normal dropout, individual nodes are dropped off the nodes in the network randomly while 1D version of the spatial dropout drops the entire 1D feature maps which helps in maintaining the independence between feature maps. The 1 × 1 conv1D layer is to ensure that the width of the output matches with the input.

### Seq-to-seq models

Sequence-to-Sequence model approach aims at mapping a fixed length input to a fixed length output. Here, input sequence and output sequence may or may not be of same length.

**Figure 5:**
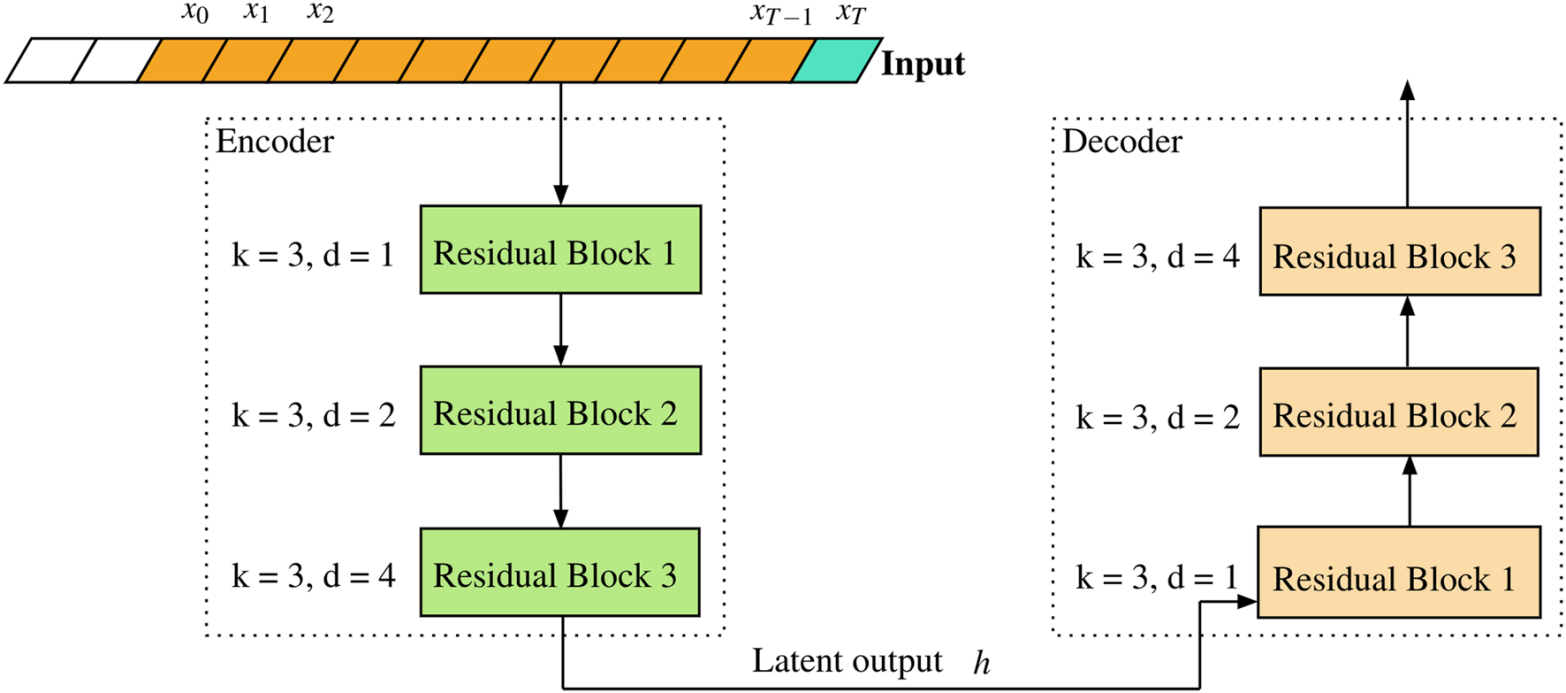
Sequence-to-sequence approach.

This approach have been popularly used in language modeling tasks such as machine translation^43^, speech recognition^44^ and video captioning^45^. The structure of a typical encoder-decoder approach involving temporal-convolutional networks is shown in Fig.  5.

Here, both the encoder and decoder consists of TCN. The encoder takes the input sequence and processes the input by passing it through different residual blocks having convolution layers with different dilation factors *d*, collecting all the relevant information and producing a encoded state vector that best summarizes or captures all the input information that were seen by the encoder TCN. This encoded state vector is also called the hidden or latent state. This hidden state is then fed to the decoder part to start the prediction of output sequence one step at a time which is then used to produce the predicted sequence output.

**Figure 6:**
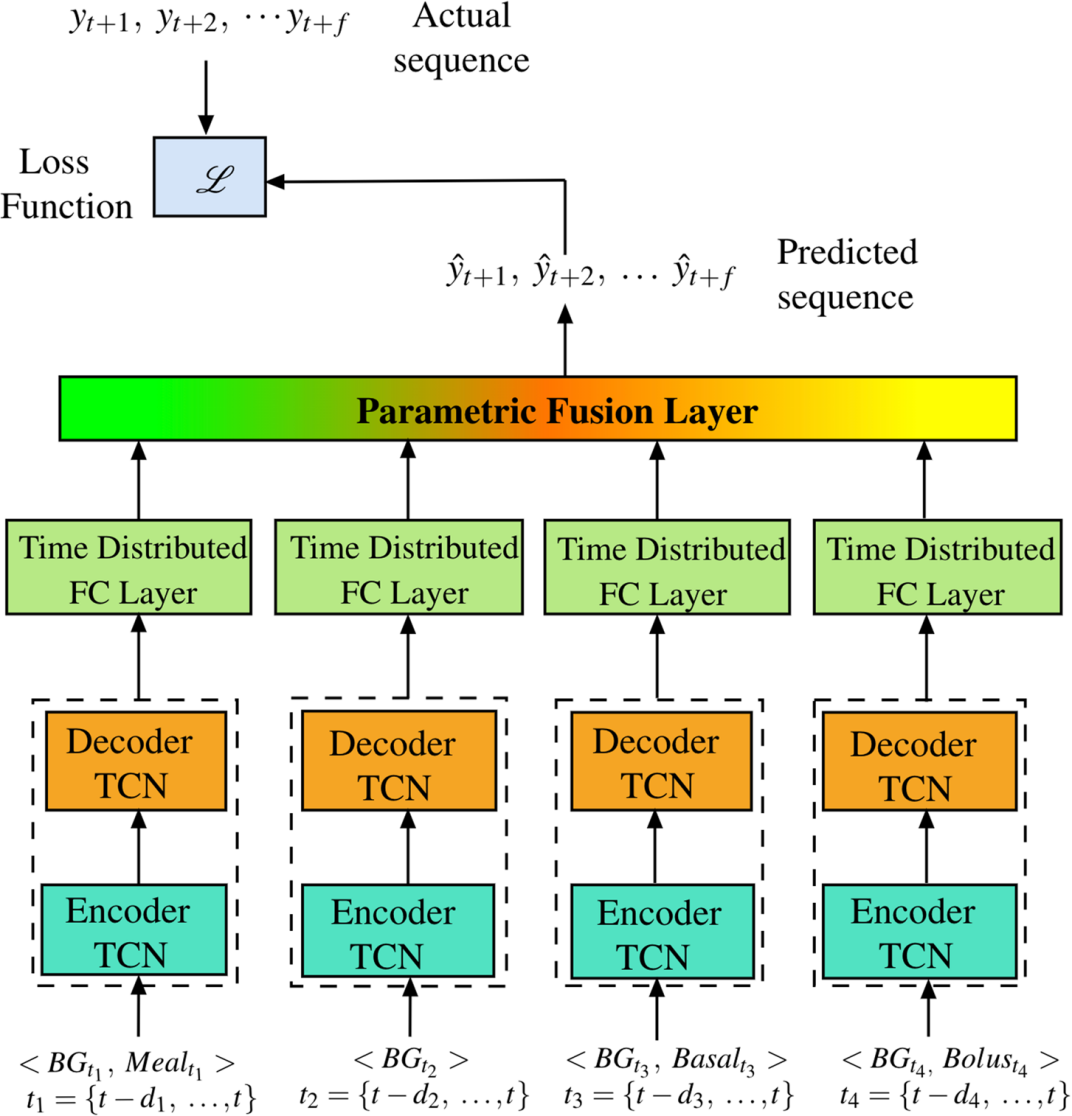
Proposed model.

### Proposed model

Our proposed model consists of four components that use temporal convolutional network embedded within encoder-decoder sequence-to-sequence model approaches that take into account different past history of input predictors as shown in Fig.  6.

Since, the model predictions are done in multi-step manner and inputs and output sequences are of variable length, we made use of encoder-decoder sequence architecture, which models the sequence to sequence tasks. The purpose of using different input time sequence is because the future blood glucose would be functionally impacted differently by different inputs. For example, the blood glucose levels for an individual with T1D are impacted by food intake (carbohydrates), basal insulin delivery, and bolus insulin administered to cover food and regulate BG levels within a target range.

Given the maximum history of available input information until timestep *t*, in order to predict BG at future timesteps until prediction horizon *f* as an output sequence, ${\hat{y}}_{t + 1}, {\hat{y}}_{t + 2}, \dots , {\hat{y}}_{t + f}$, we divide the input temporal sequence history of variables in the following four components that forms the basis of predicting the final sequence output. These *four* components are:

- **History of BG and Meal intake** In this component, we consider a longer past history of 3 h for *BG* and *meal intake* for predicting the future BG. The meal intake in the past impacts the BG levels in the future as meal or the carbohydrates intake usually increases the blood glucose levels. The input sequence for this component is $\langle \text {BG}_{t_1}, \text {Meal}_{t_1}\rangle$ where $t_1 = \{t - p_1, t - (p_1 - 1), t - (p_1 - 2) \dots , t\}$. Here, we denote the length of past history for this component by $p_1$.
- **Recent history of BG** This component comprises of the recent 30 mins history of BG. The close history information on the BG history is considered here to aid the improvement in output predictions. The input sequence for this component is $\langle \text {BG}_{t_2}\rangle$ where $t_3 = \{t - p_2, t - (p_2 - 1), t - (p_2 - 2) \dots , t\}$. Here, we denote the length of past history for this component by $p_2$.
- **History of BG and basal rate** This component comprises of inputs of 3 h of history of *BG* and *basal rate*. Basal insulin is the background insulin that runs continuously and regulates BG levels between meals, and during times of fasting (ie sleep). The input sequence for this component is $\langle \text {BG}_{t_3}, \text {Basal}_{t_3}\rangle$ where $t_3 = \{t - p_3, t - (p_3 - 1), t - (p_3 - 2), \dots , t\}$. Here, we denote the length of past history for this component by $p_3$.
- **History of BG and bolus insulin** In this component, we take the input history of 3 h for BG and bolus insulin. This insulin is required when the patient consumes a meal or gives insulin to correct a high blood sugar value. The amount of bolus insulin required by the body is largely dependent on amount of meal intake (usually in grams), total daily insulin requirements, and the target range of the BG level. The input sequence for this component is $\langle \text {BG}_{t_4}, \text {Bolus}_{t_4}\rangle$ where $t_4 = \{t - p_4, t - (p_4 - 1), t - (p_4 - 2), \dots , t\}$. Here, we denote the length of past history for this component by $p_4$.

In the model shown in Fig. 6, the starting position history for the inputs sequence length in the four components can be determine through $p_1$, $p_2$, $p_3$ and $p_4$. The predicted output sequence from these four individual components are then fed into the time distributed layer that further applies the fully-connected or dense layer to every temporal slice of the output sequence. After this, the final output sequences from the time-distributed layers are fused with a weighted parametric layer that looks for the optimum set of contributions that results in accurate future blood glucose values.

#### Parametric fusion layer

In the proposed model architecture, we have four different components that accounts for different histories of variable combinations for predicting the sequence output. We compute final predicted output sequence by fusing the sequence outputs of different components of the model with associated learnable component weighted parameters as below:

$$\begin{aligned} \hat{{\mathbf {Y}}} = {\mathbf {W}}_1 \odot \hat{{\mathbf {Y}}}_1 + {\mathbf {W}}_2 \odot \hat{{\mathbf {Y}}}_2 + {\mathbf {W}}_3 \odot \hat{{\mathbf {Y}}}_3 + {\mathbf {W}}_4 \odot \hat{{\mathbf {Y}}}_4 \end{aligned}$$

Here, $\hat{\mathbf {Y_1}}$, $\hat{\mathbf {Y_2}}$, $\hat{\mathbf {Y_3}}$ and $\hat{\mathbf {Y_4}}$ are the predicted sequence output coming out of the four component of the model while $\mathbf {W_1}, \mathbf {W_2}, \mathbf {W_3}$ and $\mathbf {W_4}$ are the trainable weight parameters that indicates the degree of influence that each of the component has on the final sequence prediction.

#### Loss function

We used Huber loss as the objective function for the proposed model. Huber loss is less sensitive to outliers unlike mean squared error. It is defined as:

$$\begin{aligned} L_\delta ({\mathbf {y}}, \hat{{\mathbf {y}}})= {\left\{ \begin{array}{ll} \frac{1}{2}({\mathbf {y}} - \hat{{\mathbf {y}}})^2,&{} \text {for } |{\mathbf {y}} - \hat{{\mathbf {y}}}|\le \delta \\ \delta (|{\mathbf {y}} - \hat{{\mathbf {y}}}| - \frac{1}{2}\delta ), &{} \text {otherwise} \end{array}\right. } \end{aligned}$$

where ${\mathbf {y}}$ and $\hat{{\mathbf {y}}}$ are the predicted and actual sequence output. Huber loss is the combination of squared loss and absolute loss in its definition. It becomes quadratic when the error is small else it is linear. The control of boundary that defines the transition from quadratic to linear is done by tuning of hyper-parameter $\delta$. For our experiments we set $\delta = 0.5$.

**Figure 7:**
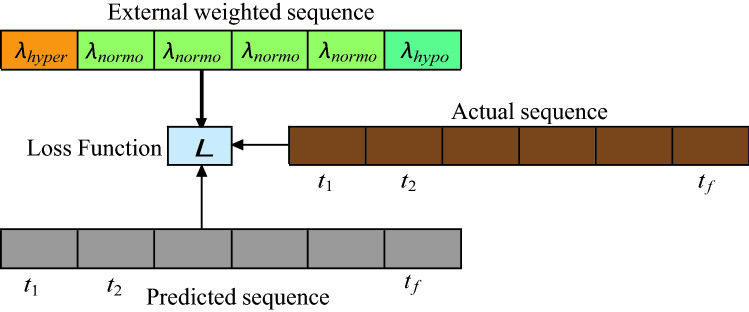
Weighted loss function.

To improve predictions, we have also incorporated the weighted values for different events. For example, we penalize the cost of incorrect hypoglycemic values by multiplying it with weight, denoted by $\lambda _{\text {hypo}}$; the cost of penalizing the incorrect hypoglycemic values is taken by multiplying it with $\lambda _{\text {hyper}}$ with the cost of incorrect normoglycemic values being denoted by $\lambda _{\text {normo}}$. The illustration of adding the additional cost to the sample sequence is shown in Fig. 7. For our experiments, we use $\lambda _{\text {hypo}} = 100$, $\lambda _{\text {hyper}} = 10$ and $\lambda _{\text {normo}} = 1$. The introduction of extra-penalty terms can cause non-smoothness in the loss function, however, with enough number of training epochs and the stated learning rate and batch size, the loss converges and is effective in improving the generalizability of the model in better capturing of the rare events such as hypoglycemia and hyperglycemia.

## Experimental set-up

The proposed model was tested on actual blood glucose data for 97 patients as discussed in Data section. The experiments were conducted on a computing cluster available through Centre for Computational Research (CCR) in University at Buffalo. The nodes were equipped with NVIDIA Tesla V100 GPUs with 16GB memory. We used *Keras* library^46^ with the *Tensorflow* library^47^ as backend.

### Data preparation

The data has been normalized using Min-Max normalization method to normalize or scale the data into the range [0, 1] before feeding into the model. The output predictions from the model are then de-normalized or re-scaled back to permit comparing and evaluating with groundtruth values using different performance measures. This is an important step since normalizing the values is required before feeding into the model while de-normalizing is required for correct evaluation of model predictions.

### Training and parameter learning

Parameters of the proposed model are learned through back propagation through time (BPTT) with *Adam* as optimizer with learning rate of 0.0001. The training batch size is set to 32. For the 1D convolution layers, the kernel size for the convolution layers is set to 2 while the number of filters is set to 64. The dilation factors *d* in these layers is changed with the following values—[1, 2, 4]. The 1D spatial dropout is set to 0.2. We switched off the use of normalization layers in the residual block. We use one stack of residual block in each of the encoder and decoder module within each of the four components of our model. Based on the sensitivity analysis of history size and impact of each input variables on the forecasting skill of the model, we have set these values as $p_1 = p_3 = p_4 = 36$ and $p_2 = 6$, since $36 * 5 = 180$ mins and $6 * 5 = 30$ mins. We choose the past history of first, third and fourth component as 3 h to account for a better long-term dependencies. The second component has the input of recent history of 30 mins for BG as the recent values have great impact on the values that are followed in the output sequence.

### Evaluation

In this subsection, we describe the evaluation measures used for evaluating the performance of our model. The performance evaluation metrics is the key indicator of the prediction power of a developed model.

#### Quantitative evaluation metrics

The performance metrics used in our experiment for evaluating the prediction accuracy of the proposed model are listed as below:

1. **Root mean squared error (RMSE)**
  $$\begin{aligned} RMSE = \sqrt{\frac{1}{N}\sum _{i=1}^{N}({\hat{y}}_i - {y}_i)^2} \end{aligned}$$
2. **Mean absolute error (MAE)**
  $$\begin{aligned} MAE = \frac{1}{N}\sum _{i=1}^{N}\vert {\hat{y}}_i - {y}_i\vert \end{aligned}$$
3. **Mean absolute percentage error (MAPE)**
  $$\begin{aligned} MAPE = \frac{1}{N} \sum _{i=1}^{N}\frac{|{\hat{y}}_i - y_i|}{y_i} * 100\% \end{aligned}$$
4. **Normalized root mean squared error (NRMSE)**
  $$\begin{aligned} NRMSE = \frac{RMSE}{y_{\max } - y_{\min }} \end{aligned}$$

Here, *N* is the number of BGC values, ${\hat{y}}_i$ and $y_i$ are the predicted and actual BGC values respectively, while $y_{\max }$ and $y_{\min }$ are the maximum and minimum of the actual BGC values respectively. These evaluation metrics describes the quantitative accuracy for blood glucose predictions. Additionally, we also report different accuracies (i.e. true positive rate, false positive rate) for hypoglycemia, hyperglycemia and normoglycemia predictions. Here, we defined True Positive Rate = TP/P while False Positive Rate = FP/N. Here, TP, P, FP and N implies the True Positives, Positives, False Positives and Negatives respectively. To determine the True positive rate and false positive rate, for hypo-, hyper- and normoglycemia, we define the relevant terms as described below:

- **Hypoglycemia** For hypoglycemia (BG < 70 mg/dL), the True Positives (TP) is defined as the number of times the predicted BG level and the corresponding actual BG level < 70 mg/dL while the Positives (P) is defined as the number of times actual BG levels < 70 mg/dL. Here, we also define the False Positives (FP) as the number of times the predicted BG level < 70 mg/dL and the corresponding actual BG level $>=$ 70 mg/dL while the Negatives (N) is defined as the number of times actual BG levels $>=$ 70 mg/dL.
- **Hyperglycemia** For hyperglycemia (BG $>=$ 180 mg/dL), the True Positives (TP) is defined as the number of times the predicted BG level and the corresponding actual BG level $>=$ 180 mg/dL while the Positives (P) is defined as the number of times actual BG levels $>=$ 180 mg/dL. Here, we also define the False Positives (FP) as the number of times the predicted BG level $>=$ 180 mg/dL and the corresponding actual BG level < 180 mg/dL while the Negatives (N) is defined as the number of times actual BG levels < 180 mg/dL.
- **Normoglycemia** For Normoglycemia (70 mg/dL $<=$ BG < 180 mg/dL), the True Positives (TP) is defined as the number of times the predicted BG level and the corresponding actual BG level $>=$ 70 mg/dL and < 180 mg/dL while the Positives (P) is defined as the number of times actual BG levels $>=$70 mg/dL and < 180 mg/dL. Here, we also define the False Positives (FP) as the number of times the predicted BG level $>=$ 70 mg/dL and < 180 mg/dL and the corresponding actual BG level < 70 mg/dL or $>=$ 180 mg/dL while the Negatives (N) is defined as the number of times actual BG levels < 70 mg/dL or $>=$ 180 mg/dL.

While all the previously mentioned metrics are good indicators of the model’s performance quantitatively, they are unable to clearly represent the qualitative performance like clinical accuracy of modeled blood glucose meter. In order to represent this qualitative measure of the model, we make use of error grid analysis which is described in the following section.

#### Error grid analysis

To measure the clinical accuracy of our model predictions, we used both the Parkes^48^ and the Clarke^49^ error grid system. In each of these grid systems, the errors are categorized into zones and each zone covers a certain range of prediction and reference values. The pairing of the corresponding values in the actual blood glucose and predicted blood glucose levels is plotted in the error grid. Each of these pairs falls in one of the zones in the error grid. Each zone represents the degree of risk of an outcome arising due to the difference in error between predicted and actual values. The performance of the model can be best assessed by having more number of pairs of predicted and actual points appearing in Zone A. Zone A is defined as the grid region where predicted values fall within ± 20% of the actual data. In clinical practice, predictions which fall within this Zone would not be expected to alter clinical action by the patient.

## Conclusion and future work

We present here a novel deep learning based model to predict future BG of T1D patients in a multi-step ahead manner. This model takes the past history of BG, insulin (basal and bolus) and meal intake in order to predict the multi-step ahead BG values. The novelty of the the proposed model lies in accounting of different view of past history for different variables like meal intake, basal rate and insulin boluses. Results with validation data for 97 T1D patients demonstrate that our proposed model performs consistently on both qualitative and quantitative metrics. Our proposed model outperforms some of the competitive and baseline approaches while it is also shown that the ARX model seems to be one of the challenging prediction model in comparison to our model. It has been shown that our model is able to capture the hyperglycemic and the hypoglycemic events better as compare to the ARX model but is slightly less accurate in the normoglycemic range values. In the future, we would incorporate either other patient-related information or make an ensemble fused modelling approach to overall enhance the prediction accuracy. The other direction can be considered in incorporating a probabilistic layer that can quantify the uncertainty of input and output.

## Supplementary Information

Supplementary Information.
