# Supplementary material for: Multi-step ahead predictive model for blood glucose concentrations of type-1 diabetic patients
Source: Sci Rep. 2021 Dec 21;11:24332. doi: 10.1038/s41598-021-03341-5 (PMC8692478; doi:10.1038/s41598-021-03341-5)
Supplement: Supplementary file 1 — Supplementary Information. [file 41598_2021_3341_MOESM1_ESM.pdf]

Multi-step ahead predictive model for Blood Glucose Concentrations of Type-1 Diabetic Patients

Supplementary Information

1 Tables

PatientID	Data type	# CGM Samples	Mean CGM	# Hypovalues	# Hypervalues	# Normovalues	# Meals taken	Mean basal	Mean bolus
1	Train	36435	98.82	1244	86	35105	696	0.06	0.05
	Test	7713	96.26	304	2	7407	126	0.06	0.04
2	Train	25636	109.92	1058	648	23930	469	0.10	0.06
	Test	7331	116.10	157	230	6944	117	0.09	0.06
3	Train	191861	96.84	16126	757	174978	5309	0.07	0.04
	Test	8615	97.33	256	2	8357	183	0.06	0.04
4	Train	92282	119.86	2578	5898	83806	2288	0.07	0.05
	Test	8577	108.85	291	174	8112	248	0.06	0.03
5	Train	25818	101.19	2783	301	22734	437	0.06	0.03
	Test	7970	98.45	985	97	6888	168	0.06	0.03
6	Train	30940	125.11	858	2793	27289	896	0.09	0.07
	Test	6956	131.79	128	853	5975	181	0.09	0.05
7	Train	61836	99.33	6346	1213	54277	755	0.08	0.02
	Test	7674	95.79	1036	11	6627	158	0.07	0.02
8	Train	44224	128.05	1136	4635	38453	517	0.26	0.20
	Test	8117	131.62	141	1045	6931	114	0.32	0.21
9	Train	77354	129.29	1768	8520	67066	2041	0.08	0.03
	Test	3132	110.68	115	40	2977	63	0.08	0.03
10	Train	38206	129.42	1040	4180	32986	560	0.10	0.05
	Test	8017	142.31	108	1418	6491	106	0.12	0.03
11	Train	25926	127.89	759	2925	22242	755	0.03	0.05
	Test	8120	129.16	212	955	6953	212	0.04	0.06
12	Train	39857	111.54	3632	2097	34128	39	0.05	0.05
	Test	7214	105.91	885	230	6099	0	0.06	0.05
13	Train	31272	123.64	1737	2991	26544	1198	0.10	0.09
	Test	8519	126.50	352	950	7217	285	0.10	0.07
14	Train	47206	127.76	1819	5382	40005	1285	0.09	0.05
	Test	8267	132.55	277	1333	6657	221	0.07	0.04
15	Train	41081	116.92	2694	3602	34785	882	0.06	0.07
	Test	8529	129.90	340	1322	6867	254	0.04	0.07
16	Train	32520	113.25	3196	1632	27692	498	0.08	0.03
	Test	7557	117.41	635	540	6382	96	0.09	0.03
17	Train	19211	127.86	413	1982	16816	292	0.10	0.06
	Test	1791	132.52	127	296	1368	29	0.11	0.08
18	Train	26460	131.97	628	3457	22375	710	0.06	0.02
	Test	4495	130.85	83	549	3863	93	0.06	0.02
19	Train	45299	133.88	1404	5780	38115	654	0.03	0.02
	Test	7625	149.47	239	1806	5580	109	0.03	0.02
20	Train	76955	125.76	3339	9018	64598	1495	0.08	0.08
	Test	8326	123.63	426	980	6920	148	0.08	0.09
21	Train	35070	125.99	2066	3670	29334	764	0.08	0.07
	Test	7523	129.26	394	936	6193	160	0.08	0.07
22	Train	26225	130.94	898	3606	21721	308	0.07	0.05
	Test	8023	127.14	265	812	6946	84	0.07	0.04
23	Train	28005	131.06	673	4204	23128	897	0.09	0.08
	Test	8549	134.50	141	1300	7108	302	0.09	0.10
24	Train	24989	132.42	841	3582	20566	463	0.07	0.06
	Test	6616	113.46	422	335	5859	107	0.06	0.05
25	Train	41086	132.42	1953	5614	33519	941	0.07	0.04
	Test	6387	127.84	437	897	5053	152	0.07	0.03
26	Train	40037	135.65	858	6638	32541	880	0.10	0.11
	Test	8007	133.43	175	1270	6562	171	0.12	0.13
27	Train	21224	121.83	1764	2130	17330	627	0.03	0.05
	Test	6712	122.71	455	713	5544	194	0.03	0.05
	Train	25963	121.36	2187	2850	20926	670	0.11	0.08

28	Test	8598	139.36	268	1583	6747	235	0.12	0.11
29	Train	20504	128.99	1136	2684	16684	432	0.05	0.04
	Test	3399	135.07	169	563	2667	62	0.05	0.04
30	Train	39075	137.75	1052	6661	31362	1041	0.06	0.08
	Test	8550	139.16	384	1610	6556	205	0.08	0.08
31	Train	32815	126.46	2246	4261	26308	708	0.01	0.01
	Test	8291	121.67	633	973	6685	225	0.01	0.01
32	Train	27505	139.36	606	5108	21791	748	0.06	0.07
	Test	7446	140.47	175	1374	5897	162	0.06	0.07
33	Train	21551	124.71	1830	2684	17037	713	0.03	0.03
	Test	7069	126.30	376	749	5944	224	0.03	0.03
34	Train	32425	137.01	1500	5492	25433	466	0.13	0.06
	Test	6574	123.15	356	673	5545	143	0.13	0.06
35	Train	24811	140.24	658	4928	19225	741	0.06	0.03
	Test	7903	128.35	145	771	6987	210	0.06	0.02
36	Train	24410	127.69	1825	3696	18889	762	0.06	0.05
	Test	7664	124.71	310	887	6467	206	0.06	0.04
37	Train	39747	136.27	2049	7081	30617	965	0.03	0.04
	Test	7239	129.64	391	879	5969	148	0.02	0.04
38	Train	39623	116.19	4843	3828	30952	1882	0.05	0.07
	Test	6466	116.49	682	464	5320	270	0.05	0.06
39	Train	56837	137.90	2441	10535	43861	1678	0.15	0.09
	Test	7709	145.31	90	1611	6008	161	0.16	0.06
40	Train	68900	146.21	866	14903	53131	917	0.37	0.10
	Test	6342	155.59	35	1653	4654	92	0.30	0.08
41	Train	37730	132.93	1878	6959	28893	412	0.09	0.07
	Test	7220	132.06	513	1049	5658	62	0.08	0.05
42	Train	62894	146.81	953	14055	47886	1514	0.03	0.03
	Test	4244	132.32	100	680	3464	98	0.03	0.03
43	Train	21768	136.69	1177	4373	16218	289	0.10	0.06
	Test	6443	143.52	334	1521	4588	76	0.11	0.06
44	Train	96267	136.69	4467	18796	73004	2154	0.04	0.04
	Test	8219	144.57	468	2361	5390	211	0.05	0.06
45	Train	24360	119.69	3122	3022	18216	808	0.03	0.04
	Test	6609	122.87	811	793	5005	226	0.03	0.03
46	Train	29484	148.72	749	7190	21545	357	0.08	0.04
	Test	5757	146.23	39	1150	4568	78	0.08	0.04
47	Train	35359	140.51	1548	7794	26017	352	0.17	0.07
	Test	7500	135.34	161	1318	6021	78	0.16	0.08
48	Train	29832	136.75	1840	6037	21955	748	0.13	0.11
	Test	8152	145.34	595	2071	5486	202	0.18	0.11
49	Train	31651	135.78	2725	5744	23182	609	0.05	0.04
	Test	5270	164.03	288	1947	3035	93	0.04	0.04
50	Train	30812	140.05	1909	6947	21956	1177	0.04	0.02
	Test	7232	141.45	472	1637	5123	247	0.03	0.02
51	Train	25247	139.74	1713	5589	17945	551	0.05	0.04
	Test	8484	121.73	801	925	6758	180	0.04	0.03
52	Train	64705	151.97	1320	18283	45102	730	0.12	0.04
	Test	4886	145.20	145	1086	3655	65	0.11	0.04
53	Train	22678	147.89	710	6011	15957	313	0.03	0.02
	Test	5211	155.28	141	1590	3480	67	0.03	0.02
54	Train	37199	152.23	1000	10400	25799	810	0.12	0.11
	Test	8117	140.81	471	1764	5882	176	0.13	0.11
55	Train	28518	158.64	279	8669	19570	720	0.06	0.06
	Test	7999	158.79	101	2223	5675	191	0.06	0.06
56	Train	28252	160.81	243	8748	19261	701	0.14	0.07
	Test	7595	155.71	65	2215	5315	211	0.14	0.07
57	Train	33359	160.58	470	10384	22505	236	0.12	0.04
	Test	7381	157.94	153	2316	4912	66	0.14	0.05
58	Train	23188	152.56	1063	6734	15391	576	0.04	0.03
	Test	7361	150.69	234	2035	5092	147	0.04	0.03
59	Train	28504	164.98	374	9937	18193	364	0.12	0.08
	Test	8072	161.09	133	2495	5444	80	0.13	0.05
60	Train	27251	155.08	1551	8287	17413	632	0.04	0.06
	Test	8245	152.65	561	2452	5232	142	0.03	0.06
61	Train	46559	162.07	931	15894	29734	857	0.07	0.05
	Test	6259	161.21	103	1878	4278	123	0.07	0.05
	Train	50593	163.87	1044	17971	31578	514	0.21	0.08

62	Test	7262	169.40	129	2977	4156	62	0.26	0.07
63	Train	87269	167.12	1181	32344	53744	1209	0.16	0.08
	Test	7549	167.53	223	2793	4533	114	0.16	0.08
64	Train	23231	175.70	448	9175	13608	504	0.12	0.11
	Test	8174	165.38	100	2783	5291	145	0.13	0.09
65	Train	26516	168.69	943	9764	15809	523	0.13	0.09
	Test	8147	153.92	421	2346	5380	123	0.14	0.09
66	Train	39462	177.61	115	17173	22174	355	0.11	0.02
	Test	6567	159.33	114	2115	4338	55	0.10	0.02
67	Train	36992	184.90	351	15848	20793	444	0.13	0.05
	Test	7910	178.95	157	3370	4383	75	0.14	0.04
68	Train	24196	180.04	437	10320	13439	606	0.23	0.08
	Test	3801	166.67	132	1336	2333	59	0.26	0.06
69	Train	45438	181.91	250	20028	25160	377	0.25	0.11
	Test	7356	185.69	41	3514	3801	6	0.26	0.09
70	Train	26230	179.25	94	11432	14704	241	0.12	0.08
	Test	7306	191.56	52	3669	3585	56	0.12	0.09
71	Train	23344	174.36	758	10090	12496	446	0.07	0.22
	Test	7029	178.10	228	3099	3702	111	0.08	0.20
72	Train	40391	200.99	14	21269	19108	812	0.06	0.06
	Test	8513	190.90	20	3898	4595	160	0.06	0.06
73	Train	100573	220.93	0	78978	21595	1667	0.10	0.01
	Test	8585	215.42	0	6425	2160	137	0.10	0.01
74	Train	30853	123.95	150	1379	29324	422	0.09	0.09
	Test	8221	122.40	31	288	7902	85	0.12	0.07
75	Train	23722	121.47	212	1212	22298	684	0.07	0.06
	Test	6964	126.90	61	518	6385	190	0.08	0.07
76	Train	26941	125.05	158	1810	24973	563	0.01	0.01
	Test	7780	124.31	28	548	7204	171	0.02	0.02
77	Train	39227	131.40	132	3709	35386	995	0.10	0.09
	Test	8551	132.49	34	1067	7450	198	0.11	0.09
78	Train	113935	126.16	1667	9423	102845	2138	0.06	0.02
	Test	7271	130.01	93	754	6424	138	0.07	0.01
79	Train	37338	130.58	184	3533	33621	787	0.09	0.06
	Test	8570	128.98	124	893	7553	174	0.09	0.07
80	Train	45400	138.63	132	5830	39438	859	0.03	0.03
	Test	8124	140.21	18	991	7115	186	0.03	0.03
81	Train	35787	138.67	53	4702	31032	795	0.08	0.10
	Test	8179	136.50	4	969	7206	202	0.08	0.09
82	Train	22219	132.59	407	2928	18884	404	0.05	0.09
	Test	2400	134.56	28	282	2090	46	0.06	0.09
83	Train	20595	143.27	70	3054	17471	324	0.08	0.04
	Test	6777	139.73	27	778	5972	120	0.08	0.04
84	Train	121153	142.21	658	16901	103594	1360	0.10	0.02
	Test	8357	139.47	24	954	7379	136	0.09	0.03
85	Train	27070	134.36	433	3399	23238	714	0.06	0.04
	Test	7861	146.43	98	1727	6036	194	0.06	0.04
86	Train	61391	134.86	930	8272	52189	1198	0.07	0.05
	Test	8459	129.80	182	916	7361	158	0.08	0.05
87	Train	38491	136.03	465	5780	32246	866	0.09	0.07
	Test	8234	141.33	50	1280	6904	178	0.10	0.08
88	Train	30025	136.74	386	4787	24852	654	0.10	0.08
	Test	8334	132.55	100	1105	7129	190	0.09	0.08
89	Train	28744	137.87	331	4601	23812	508	0.16	0.09
	Test	8484	121.45	109	542	7833	115	0.13	0.06
90	Train	34318	141.73	422	5963	27933	445	0.07	0.04
	Test	8519	135.44	126	1032	7361	131	0.08	0.05
91	Train	38140	145.79	288	6815	31037	844	0.09	0.06
	Test	8534	142.99	84	1446	7004	188	0.10	0.07
92	Train	28418	153.43	31	5476	22911	383	0.13	0.12
	Test	8555	151.77	0	1592	6963	132	0.15	0.13
93	Train	14186	146.87	238	3015	10933	234	0.12	0.07
	Test	4246	151.98	19	1089	3138	81	0.13	0.08
94	Train	73299	149.74	681	17187	55431	1379	0.06	0.07
	Test	7846	159.24	35	2212	5599	173	0.07	0.06
95	Train	36945	154.90	198	9326	27421	575	0.09	0.04
	Test	8298	146.71	81	1624	6593	143	0.09	0.03
	Train	30845	154.13	330	8130	22385	677	0.11	0.09

96	Test	7910	156.82	70	2177	5663	180	0.10	0.08
97	Train	78904	157.64	110	22811	55983	1464	0.10	0.09
	Test	8533	159.80	9	2661	5863	173	0.10	0.10

Supplementary Table S1. Patient data characteristics

PatientID	Data type	Duration (in days)	Std CGM	CV CGM	Time in hypo (in hours)	Time in hyper (in hours)	Time in normo (in hours)
1	Train	126.44	18.28	0.19	103.67	7.17	2925.42
	Test	26.77	16.70	0.17	25.33	0.17	617.25
2	Train	88.97	28.99	0.26	88.17	54.00	1994.17
	Test	25.44	29.05	0.25	13.08	19.17	578.67
3	Train	665.81	21.94	0.23	1343.83	63.08	14581.50
	Test	29.91	17.69	0.18	21.33	0.17	696.42
4	Train	320.30	35.31	0.29	214.83	491.50	6983.83
	Test	29.77	27.53	0.25	24.25	14.50	676.00
5	Train	89.59	27.33	0.27	231.92	25.08	1894.50
	Test	27.66	26.16	0.27	82.08	8.08	574.00
6	Train	107.38	40.08	0.32	71.50	232.75	2274.08
	Test	24.14	42.55	0.32	10.67	71.08	497.92
7	Train	214.60	28.52	0.29	528.83	101.08	4523.08
	Test	26.64	22.95	0.24	86.33	0.92	552.25
8	Train	153.49	39.48	0.31	94.67	386.25	3204.42
	Test	28.17	43.43	0.33	11.75	87.08	577.58
9	Train	268.38	40.96	0.32	147.33	710.00	5588.83
	Test	10.86	29.64	0.27	9.58	3.33	248.08
10	Train	132.61	38.88	0.30	86.67	348.33	2748.83
	Test	27.83	41.32	0.29	9.00	118.17	540.92
11	Train	89.98	43.29	0.34	63.25	243.75	1853.50
	Test	28.18	46.23	0.36	17.67	79.58	579.42
12	Train	138.34	36.82	0.33	302.67	174.75	2844.00
	Test	25.04	34.13	0.32	73.75	19.17	508.25
13	Train	108.51	44.11	0.36	144.75	249.25	2212.00
	Test	29.56	43.44	0.34	29.33	79.17	601.42
14	Train	163.84	42.13	0.33	151.58	448.50	3333.75
	Test	28.69	49.16	0.37	23.08	111.08	554.75
15	Train	142.59	40.85	0.35	224.50	300.17	2898.75
	Test	29.60	46.72	0.36	28.33	110.17	572.25
16	Train	112.84	36.97	0.33	266.33	136.00	2307.67
	Test	26.23	44.20	0.38	52.92	45.00	531.83
17	Train	66.64	37.86	0.30	34.42	165.17	1401.33
	Test	6.22	52.42	0.40	10.58	24.67	114.00
18	Train	91.82	42.60	0.32	52.33	288.08	1864.58
	Test	15.60	38.40	0.29	6.92	45.75	321.92
19	Train	157.20	40.80	0.30	117.00	481.67	3176.25
	Test	26.46	56.86	0.38	19.92	150.50	465.00
20	Train	267.00	45.40	0.36	278.25	751.50	5383.17
	Test	28.89	47.92	0.39	35.50	81.67	576.67
21	Train	121.70	42.93	0.34	172.17	305.83	2444.50
	Test	26.10	47.00	0.36	32.83	78.00	516.08
22	Train	91.02	46.15	0.35	74.83	300.50	1810.08
	Test	27.85	40.33	0.32	22.08	67.67	578.83
23	Train	97.20	46.67	0.36	56.08	350.33	1927.33
	Test	29.67	51.10	0.38	11.75	108.33	592.33
24	Train	86.70	45.76	0.35	70.08	298.50	1713.83
	Test	22.97	35.06	0.31	35.17	27.92	488.25
25	Train	142.59	44.28	0.33	162.75	467.83	2793.25
	Test	22.17	45.03	0.35	36.42	74.75	421.08
26	Train	138.92	49.86	0.37	71.50	553.17	2711.75
	Test	27.79	46.41	0.35	14.58	105.83	546.83
27	Train	73.63	46.86	0.38	147.00	177.50	1444.17
	Test	23.30	47.25	0.39	37.92	59.42	462.00
28	Train	90.09	47.48	0.39	182.25	237.50	1743.83
	Test	29.85	48.02	0.34	22.33	131.92	562.25
29	Train	71.12	46.68	0.36	94.67	223.67	1390.33
	Test	11.80	46.10	0.34	14.08	46.92	222.25
30	Train	135.62	48.57	0.35	87.67	555.08	2613.50
	Test	29.67	47.54	0.34	32.00	134.17	546.33

31	Train	113.89	46.27	0.37	187.17	355.08	2192.33
	Test	28.77	44.49	0.37	52.75	81.08	557.08
32	Train	95.47	45.54	0.33	50.50	425.67	1815.92
	Test	25.84	48.36	0.34	14.58	114.50	491.42
33	Train	74.77	48.50	0.39	152.50	223.67	1419.75
	Test	24.52	45.19	0.36	31.33	62.42	495.33
34	Train	112.52	51.55	0.38	125.00	457.67	2119.42
	Test	22.81	43.30	0.35	29.67	56.08	462.08
35	Train	86.10	49.74	0.35	54.83	410.67	1602.08
	Test	27.43	36.95	0.29	12.08	64.25	582.25
36	Train	84.71	54.74	0.43	152.08	308.00	1574.08
	Test	26.60	42.96	0.34	25.83	73.92	538.92
37	Train	137.92	49.25	0.36	170.75	590.08	2551.42
	Test	25.12	45.72	0.35	32.58	73.25	497.42
38	Train	137.49	48.27	0.42	403.58	319.00	2579.33
	Test	22.44	40.98	0.35	56.83	38.67	443.33
39	Train	197.24	52.51	0.38	203.42	877.92	3655.08
	Test	26.75	46.99	0.32	7.50	134.25	500.67
40	Train	239.04	48.56	0.33	72.17	1241.92	4427.58
	Test	22.01	53.81	0.35	2.92	137.75	387.83
41	Train	130.94	53.40	0.40	156.50	579.92	2407.75
	Test	25.06	56.79	0.43	42.75	87.42	471.50
42	Train	218.22	50.09	0.34	79.42	1171.25	3990.50
	Test	14.73	43.06	0.33	8.33	56.67	288.67
43	Train	75.54	60.24	0.44	98.08	364.42	1351.50
	Test	22.36	63.71	0.44	27.83	126.75	382.33
44	Train	334.10	54.79	0.40	372.25	1566.33	6083.67
	Test	28.53	63.20	0.44	39.00	196.75	449.17
45	Train	84.51	49.16	0.41	260.17	251.83	1518.00
	Test	22.94	49.50	0.40	67.58	66.08	417.08
46	Train	102.30	50.72	0.34	62.42	599.17	1795.42
	Test	19.98	45.21	0.31	3.25	95.83	380.67
47	Train	122.70	52.75	0.38	129.00	649.50	2168.08
	Test	26.03	51.60	0.38	13.42	109.83	501.75
48	Train	103.52	58.97	0.43	153.33	503.08	1829.58
	Test	28.29	64.94	0.45	49.58	172.58	457.17
49	Train	109.81	56.66	0.42	227.08	478.67	1931.83
	Test	18.28	76.24	0.46	24.00	162.25	252.92
50	Train	106.93	56.84	0.41	159.08	578.92	1829.67
	Test	25.10	55.72	0.39	39.33	136.42	426.92
51	Train	87.62	60.24	0.43	142.75	465.75	1495.42
	Test	29.45	48.23	0.40	66.75	77.08	563.17
52	Train	224.52	55.17	0.36	110.00	1523.58	3758.50
	Test	16.95	51.63	0.36	12.08	90.50	304.58
53	Train	78.71	57.47	0.39	59.17	500.92	1329.75
	Test	18.09	59.68	0.38	11.75	132.50	290.00
54	Train	129.09	60.87	0.40	83.33	866.67	2149.92
	Test	28.17	60.88	0.43	39.25	147.00	490.17
55	Train	98.98	52.72	0.33	23.25	722.42	1630.83
	Test	27.77	59.75	0.38	8.42	185.25	472.92
56	Train	98.06	54.65	0.34	20.25	729.00	1605.08
	Test	26.36	53.20	0.34	5.42	184.58	442.92
57	Train	115.77	59.99	0.37	39.17	865.33	1875.42
	Test	25.61	59.68	0.38	12.75	193.00	409.33
58	Train	80.47	58.40	0.38	88.58	561.17	1282.58
	Test	25.54	55.40	0.37	19.50	169.58	424.33
59	Train	98.93	58.36	0.35	31.17	828.08	1516.08
	Test	28.02	61.78	0.38	11.08	207.92	453.67
60	Train	94.57	67.74	0.44	129.25	690.58	1451.08
	Test	28.62	66.97	0.44	46.75	204.33	436.00
61	Train	161.58	55.01	0.34	77.58	1324.50	2477.83
	Test	21.72	46.60	0.29	8.58	156.50	356.50
62	Train	175.60	64.42	0.39	87.00	1497.58	2631.50
	Test	25.20	65.65	0.39	10.75	248.08	346.33
63	Train	302.83	62.04	0.37	98.42	2695.33	4478.67
	Test	26.19	67.80	0.40	18.58	232.75	377.75
	Train	80.62	75.15	0.43	37.33	764.58	1134.00

64	Test	28.36	72.14	0.44	8.33	231.92	440.92
65	Train	92.05	67.79	0.40	78.58	813.67	1317.42
	Test	28.28	61.74	0.40	35.08	195.50	448.33
66	Train	136.94	59.16	0.33	9.58	1431.08	1847.83
	Test	22.79	56.91	0.36	9.50	176.25	361.50
67	Train	128.36	69.09	0.37	29.25	1320.67	1732.75
	Test	27.45	69.48	0.39	13.08	280.83	365.25
68	Train	83.94	71.73	0.40	36.42	860.00	1119.92
	Test	13.18	74.48	0.45	11.00	111.33	194.42
69	Train	157.68	66.10	0.36	20.83	1669.00	2096.67
	Test	25.53	65.02	0.35	3.42	292.83	316.75
70	Train	91.03	58.23	0.32	7.83	952.67	1225.33
	Test	25.36	67.68	0.35	4.33	305.75	298.75
71	Train	81.00	68.05	0.39	63.17	840.83	1041.33
	Test	24.39	68.60	0.39	19.00	258.25	308.50
72	Train	140.20	68.18	0.34	1.17	1772.42	1592.33
	Test	29.56	66.81	0.35	1.67	324.83	382.92
73	Train	349.06	51.16	0.23	0.00	6581.50	1799.58
	Test	29.80	49.76	0.23	0.00	535.42	180.00
74	Train	107.09	28.44	0.23	12.50	114.92	2443.67
	Test	28.53	27.28	0.22	2.58	24.00	658.50
75	Train	82.33	31.20	0.26	17.67	101.00	1858.17
	Test	24.17	32.94	0.26	5.08	43.17	532.08
76	Train	93.50	34.12	0.27	13.17	150.83	2081.08
	Test	27.00	32.37	0.26	2.33	45.67	600.33
77	Train	136.15	33.33	0.25	11.00	309.08	2948.83
	Test	29.68	36.48	0.28	2.83	88.92	620.83
78	Train	395.40	37.05	0.29	138.92	785.25	8570.42
	Test	25.23	39.66	0.31	7.75	62.83	535.33
79	Train	129.61	35.13	0.27	15.33	294.42	2801.75
	Test	29.75	37.77	0.29	10.33	74.42	629.42
80	Train	157.60	35.24	0.25	11.00	485.83	3286.50
	Test	28.20	33.57	0.24	1.50	82.58	592.92
81	Train	124.20	39.04	0.28	4.42	391.83	2586.00
	Test	28.39	40.24	0.29	0.33	80.75	600.50
82	Train	77.07	43.54	0.33	33.92	244.00	1573.67
	Test	8.32	38.33	0.28	2.33	23.50	174.17
83	Train	71.45	36.14	0.25	5.83	254.50	1455.92
	Test	23.51	35.59	0.25	2.25	64.83	497.67
84	Train	420.48	36.97	0.26	54.83	1408.42	8632.83
	Test	29.01	34.26	0.25	2.00	79.50	614.92
85	Train	93.95	39.99	0.30	36.08	283.25	1936.50
	Test	27.28	46.56	0.32	8.17	143.92	503.00
86	Train	213.07	40.99	0.30	77.50	689.33	4349.08
	Test	29.36	37.98	0.29	15.17	76.33	613.42
87	Train	133.59	42.38	0.31	38.75	481.67	2687.17
	Test	28.58	44.76	0.32	4.17	106.67	575.33
88	Train	104.23	44.32	0.32	32.17	398.92	2071.00
	Test	28.93	41.93	0.32	8.33	92.08	594.08
89	Train	99.75	45.37	0.33	27.58	383.42	1984.33
	Test	29.44	33.50	0.28	9.08	45.17	652.75
90	Train	119.11	44.65	0.32	35.17	496.92	2327.75
	Test	29.57	38.05	0.28	10.50	86.00	613.42
91	Train	132.37	40.41	0.28	24.00	567.92	2586.42
	Test	29.62	44.97	0.31	7.00	120.50	583.67
92	Train	98.64	38.11	0.25	2.58	456.33	1909.25
	Test	29.69	35.14	0.23	0.00	132.67	580.25
93	Train	49.22	45.33	0.31	19.83	251.25	911.08
	Test	14.73	43.31	0.28	1.58	90.75	261.50
94	Train	254.41	47.98	0.32	56.75	1432.25	4619.25
	Test	27.23	43.68	0.27	2.92	184.33	466.58
95	Train	128.23	47.08	0.30	16.50	777.17	2285.08
	Test	28.80	45.23	0.31	6.75	135.33	549.42
96	Train	107.06	52.03	0.34	27.50	677.50	1865.42
	Test	27.45	51.70	0.33	5.83	181.42	471.92
97	Train	273.86	50.16	0.32	9.17	1900.92	4665.25
	Test	29.61	50.08	0.31	0.75	221.75	488.58

Supplementary Table S2. (Revised) Patient data characteristics

PID	PH=30 min.				PH=60 min.			
	RMSE	MAE	MAPE	NRMSE	RMSE	MAE	MAPE	NRMSE
74	17.71	12.22	9.86	0.08	28.84	19.82	15.57	0.14
75	17.78	12.42	9.90	0.07	29.63	20.98	16.46	0.12
76	20.77	14.80	11.70	0.11	30.71	22.04	17.20	0.16
77	14.80	10.20	7.63	0.07	25.72	18.67	14.35	0.12
78	17.10	11.88	9.34	0.06	27.18	19.51	15.71	0.09
79	20.92	14.49	11.55	0.08	33.07	23.58	18.40	0.12
80	18.70	13.39	9.67	0.08	28.83	21.93	16.15	0.13
81	18.25	13.08	9.69	0.06	30.54	22.09	16.56	0.10
82	17.00	12.45	9.58	0.08	29.09	21.92	16.68	0.14
83	18.71	11.99	8.85	0.07	28.13	19.29	14.54	0.11
84	20.30	14.41	10.65	0.07	33.00	23.94	18.12	0.11
85	24.46	17.81	12.45	0.08	40.17	31.15	22.54	0.14
86	19.15	13.52	10.73	0.09	42.82	31.99	23.56	0.19
87	16.47	11.32	8.33	0.05	31.22	22.29	16.49	0.09
88	18.19	13.00	10.22	0.07	29.46	21.73	17.34	0.11
89	16.69	11.61	9.56	0.09	26.11	19.40	16.00	0.13
90	23.28	16.35	12.08	0.10	37.63	27.34	19.86	0.16
91	19.61	14.14	10.39	0.06	31.45	22.99	16.80	0.10
92	11.76	8.20	5.42	0.06	21.60	16.79	11.58	0.10
93	21.81	14.90	10.11	0.08	33.27	23.29	16.35	0.12
94	22.08	15.29	10.24	0.08	39.57	29.50	20.09	0.15
95	19.71	13.11	9.22	0.06	34.22	23.84	16.35	0.10
96	27.87	19.43	12.68	0.09	47.06	34.94	23.60	0.14
97	17.22	12.27	8.12	0.07	30.27	22.72	15.18	0.12

Supplementary Table S3. Summary of Results for 24 patients with respect to Prediction Horizon (PH) of 30 min. and 60 min.

Component #	Input Features				Past History (in minutes)						
	Meal	BG	Basal	Bolus	p = 0	p = 30	p = 60	p = 90	p = 120	p = 150	p = 180
1	×	×	–	–	14.70	–	–	–	–	–	–
	×	√	–	–	–	14.37	14.33	14.90	14.44	14.48	14.61
	√	×	–	–	–	13.59	13.62	13.41	13.56	13.62	13.58
	√	√	–	–	–	13.39	13.45	12.92	13.30	13.95	13.71
2	–	×	–	–	13.94	–	–	–	–	–	–
	–	√	–	–	–	13.29	14.41	13.72	13.11	13.69	13.71
3	–	×	×	–	14.01	–	–	–	–	–	–
	–	×	√	–	–	13.64	13.38	13.56	13.34	13.11	13.63
	–	√	×	–	–	13.61	13.66	13.91	13.17	13.97	13.54
	–	√	√	–	–	13.21	13.44	13.71	14.42	13.67	13.71
4	–	×	–	×	13.92	–	–	–	–	–	–
	–	×	–	√	–	14.09	14.21	14.00	13.58	14.06	13.67
	–	√	–	×	–	13.93	12.68	13.48	13.89	13.35	13.41
	–	√	–	√	–	13.95	13.29	13.43	13.48	14.17	13.71

Supplementary Table S4. Sensitivity Analysis on the impact of input features with their past history (p), in minutes, on the model's performance in terms of RMSE (in mg/dL) for prediction horizon (PH) of 30 minutes; × indicates not using the feature while √ indicates the use of feature.

PID	First component						Second component						Third component						Fourth component					
	f=5	f=10	f=15	f=20	f=25	f=30	f=5	f=10	f=15	f=20	f=25	f=30	f=5	f=10	f=15	f=20	f=25	f=30	f=5	f=10	f=15	f=20	f=25	f=30
74	0.16	0.33	0.42	0.50	0.54	0.52	0.12	0.37	0.65	0.51	0.12	0.45	0.61	0.48	0.34	0.32	0.29	0.24	0.04	0.29	0.26	0.39	0.17	0.23
75	0.24	0.41	0.45	0.54	0.56	0.44	0.20	0.49	0.74	0.58	0.10	0.46	0.64	0.51	0.33	0.35	0.31	0.19	0.21	0.53	0.19	0.42	0.06	0.27
76	0.23	0.40	0.45	0.53	0.54	0.38	0.12	0.38	0.65	0.52	0.10	0.40	0.63	0.50	0.33	0.33	0.27	0.15	0.10	0.45	0.20	0.40	0.05	0.26
77	0.27	0.44	0.44	0.56	0.57	0.34	0.21	0.52	0.77	0.62	0.14	0.43	0.65	0.52	0.32	0.34	0.32	0.13	0.33	0.62	0.16	0.46	-0.008	0.27
78	0.13	0.28	0.42	0.51	0.54	0.52	0.06	0.24	0.50	0.42	0.03	0.38	0.57	0.48	0.35	0.33	0.31	0.28	0.06	0.23	0.30	0.43	0.26	0.31
79	0.06	0.29	0.26	0.39	0.48	0.25	0.20	0.53	0.74	0.61	0.24	0.39	0.48	0.25	-0.08	-0.06	0.01	-0.004	0.11	0.08	-0.11	0.12	-0.10	0.03
80	0.14	0.31	0.43	0.50	0.57	0.56	0.07	0.32	0.63	0.49	0.06	0.42	0.60	0.47	0.34	0.33	0.31	0.26	0.09	0.24	0.32	0.42	0.25	0.28
81	0.22	0.38	0.44	0.53	0.58	0.49	0.19	0.47	0.72	0.58	0.11	0.51	0.65	0.51	0.32	0.34	0.30	0.23	0.18	0.52	0.27	0.43	0.13	0.22
82	0.21	0.37	0.45	0.53	0.54	0.48	0.16	0.42	0.67	0.54	0.07	0.45	0.63	0.49	0.33	0.33	0.28	0.21	0.06	0.37	0.28	0.43	0.19	0.26
83	0.18	0.34	0.44	0.51	0.57	0.53	0.17	0.45	0.70	0.56	0.10	0.49	0.65	0.50	0.32	0.34	0.33	0.24	0.10	0.41	0.28	0.42	0.18	0.26
84	0.21	0.37	0.44	0.53	0.54	0.47	0.12	0.37	0.65	0.52	0.11	0.45	0.60	0.47	0.31	0.32	0.29	0.24	0.03	0.32	0.21	0.41	0.20	0.28
85	0.23	0.41	0.45	0.54	0.55	0.37	0.16	0.42	0.67	0.54	0.09	0.40	0.62	0.49	0.30	0.31	0.25	0.14	0.17	0.51	0.21	0.42	0.09	0.26
86	0.13	0.28	0.42	0.50	0.54	0.56	0.03	0.26	0.56	0.49	0.12	0.43	0.59	0.46	0.34	0.32	0.30	0.25	-0.003	0.11	0.20	0.33	0.25	0.33
87	0.20	0.37	0.44	0.52	0.54	0.47	0.16	0.43	0.67	0.55	0.08	0.46	0.62	0.48	0.32	0.33	0.30	0.25	0.11	0.42	0.24	0.41	0.15	0.25
88	0.19	0.34	0.41	0.50	0.53	0.48	0.16	0.43	0.67	0.53	0.10	0.45	0.62	0.49	0.33	0.32	0.31	0.25	0.08	0.37	0.26	0.39	0.17	0.24
89	0.20	0.35	0.44	0.51	0.57	0.48	0.15	0.40	0.66	0.51	0.06	0.44	0.62	0.48	0.32	0.32	0.30	0.26	0.11	0.33	0.32	0.41	0.23	0.31
90	0.05	0.24	0.24	0.36	0.48	0.26	0.18	0.49	0.71	0.56	0.22	0.38	0.44	0.35	-0.04	-0.07	0.04	-0.05	0.30	-0.11	0.06	0.08	-0.14	0.20
91	0.22	0.38	0.46	0.52	0.56	0.49	0.16	0.46	0.71	0.57	0.09	0.50	0.65	0.51	0.33	0.34	0.30	0.24	0.20	0.50	0.34	0.44	0.18	0.22
92	0.21	0.36	0.43	0.51	0.57	0.50	0.16	0.45	0.71	0.57	0.11	0.50	0.63	0.49	0.31	0.32	0.31	0.26	0.12	0.45	0.27	0.41	0.16	0.23
93	0.23	0.49	0.44	0.57	0.58	0.27	0.15	0.55	0.73	0.61	0.16	0.34	0.57	0.53	0.30	0.34	0.31	0.06	0.44	0.65	0.19	0.46	0.01	0.25
94	0.12	0.27	0.40	0.47	0.54	0.52	0.09	0.29	0.57	0.48	0.10	0.39	0.57	0.46	0.34	0.34	0.32	0.28	0.05	0.19	0.29	0.42	0.30	0.35
95	0.08	0.24	0.31	0.40	0.47	0.35	0.20	0.51	0.71	0.56	0.21	0.38	0.46	0.30	-0.02	-0.04	0.009	0.005	-0.09	0.22	0.10	0.17	0.09	0.16
96	0.21	0.38	0.45	0.54	0.57	0.50	0.17	0.47	0.72	0.58	0.11	0.49	0.64	0.50	0.32	0.34	0.29	0.23	0.17	0.49	0.26	0.46	0.19	0.29
97	0.12	0.26	0.39	0.50	0.53	0.56	0.13	0.32	0.49	0.55	0.42	0.50	0.61	0.48	0.37	0.30	0.31	0.27	0.03	0.15	0.23	0.35	0.31	0.37

Supplementary Table S5. Contribution of weights for 24 patients with respect to Prediction Horizon (PH) of 30 min.

Patient ID	Measures	Overall	Hypoglycemia	Hyperglycemia	Normoglycemia
1	RMSE	13.61	16.13	No test samples	13.50
	MAE	10.09	11.75	No test samples	10.03
	MAPE	10.46	19.70	No test samples	10.10
	NRMSE	0.11	0.13	No test samples	0.10
	TP/P	–	116/288	No test samples	6955/7261
	FP/N	–	304/7261	2/7549	172/288
2	RMSE	17.19	19.89	25.84	16.76
	MAE	12.58	14.48	20.31	12.28
	MAPE	10.90	23.67	10.38	10.66
	NRMSE	0.09	0.11	0.14	0.09
	TP/P	–	52/136	124/230	6541/6842
	FP/N	–	262/7072	262/7072	190/366
3	RMSE	11.61	14.28	63.92	11.48
	MAE	8.10	9.27	62.77	8.05
	MAPE	8.48	15.39	34.25	8.26
	NRMSE	0.09	0.11	0.47	0.09
	TP/P	–	129/248	0/2	7409/8283
	FP/N	–	870/8285	870/8285	121/250
4	RMSE	14.41	13.13	19.01	14.33
	MAE	10.13	8.54	15.34	10.07
	MAPE	9.44	13.48	7.77	9.34
	NRMSE	0.07	0.07	0.10	0.07
	TP/P	–	160/271	107/173	7570/8010
	FP/N	–	416/8183	416/8183	177/444
5	RMSE	14.81	8.66	42.17	14.71
	MAE	10.33	6.03	28.96	10.64
	MAPE	10.26	10.34	14.66	10.18
	NRMSE	0.08	0.05	0.22	0.08
	TP/P	–	781/913	50/97	6206/6755
	FP/N	–	530/6852	530/6852	179/1010
6	RMSE	20.68	18.74	27.21	19.63
	MAE	15.34	12.10	20.88	14.63
	MAPE	12.07	18.84	9.77	12.27
	NRMSE	0.07	0.07	0.09	0.07
	TP/P	–	40/104	587/814	5374/5833
	FP/N	–	229/6647	229/6647	291/918
7	RMSE	12.00	8.88	18.43	12.40
	MAE	8.70	5.92	17.70	9.12
	MAPE	9.30	10.05	9.51	9.18
	NRMSE	0.08	0.06	0.12	0.08
	TP/P	–	865/1016	0/11	5598/6524
	FP/N	–	922/6535	922/6535	162/1027
8	RMSE	16.64	9.12	23.53	15.51
	MAE	11.62	5.85	17.26	10.91
	MAPE	8.90	8.97	8.28	8.99
	NRMSE	0.05	0.03	0.07	0.05
	TP/P	–	87/141	775/998	6421/6814
	FP/N	–	118/7812	118/7812	277/1139
9	RMSE	13.48	12.51	25.43	13.27
	MAE	10.03	8.84	20.29	9.94
	MAPE	9.48	14.98	10.45	9.25
	NRMSE	0.08	0.07	0.15	0.08
	TP/P	–	48/115	22/40	2676/2854
	FP/N	–	156/2894	156/2894	85/155
10	RMSE	18.75	11.23	23.84	17.60
	MAE	13.95	7.69	18.97	12.99
	MAPE	10.10	13.84	9.09	10.25
	NRMSE	0.06	0.03	0.07	0.05
	TP/P	–	72/91	911/1346	6015/6457
	FP/N	–	211/7803	211/7803	454/1437
11	RMSE	22.14	19.10	30.71	20.75
	MAE	15.68	13.43	22.40	14.81
	MAPE	12.61	21.52	10.31	12.66
	NRMSE	0.06	0.05	0.09	0.06
	TP/P	–	101/207	718/955	6267/6835
	FP/N	–	399/7790	399/7790	343/1162

12	RMSE	23.17	12.62	30.50	24.03
	MAE	17.15	8.59	22.35	18.21
	MAPE	16.84	15.09	10.96	17.31
	NRMSE	0.10	0.05	0.13	0.10
	TP/P	–	807/878	145/224	4071/5989
	FP/N	–	1847/6213	1847/6213	149/1102
13	RMSE	17.57	10.30	24.42	16.74
	MAE	12.21	6.64	16.99	11.85
	MAPE	10.10	10.99	7.88	10.35
	NRMSE	0.06	0.03	0.08	0.05
	TP/P	–	273/337	720/928	6203/7008
	FP/N	–	678/7936	678/7936	270/1265
14	RMSE	22.92	10.86	26.07	22.60
	MAE	17.30	7.65	19.47	17.25
	MAPE	13.84	13.05	8.93	14.83
	NRMSE	0.07	0.03	0.07	0.06
	TP/P	–	203/256	984/1286	5525/6561
	FP/N	–	916/7847	916/7847	355/1542
15	RMSE	27.88	8.54	40.77	25.33
	MAE	19.84	6.35	31.30	18.30
	MAPE	15.08	10.57	14.81	15.36
	NRMSE	0.10	0.03	0.15	0.09
	TP/P	–	262/334	895/1298	5803/6733
	FP/N	–	615/8031	615/8031	473/1632
16	RMSE	19.82	11.94	30.44	19.25
	MAE	14.08	8.79	23.18	13.79
	MAPE	12.37	15.61	10.37	12.24
	NRMSE	0.05	0.03	0.08	0.05
	TP/P	–	445/590	384/540	5505/6263
	FP/N	–	656/6803	656/6803	299/1130
17	RMSE	32.56	29.04	42.36	30.28
	MAE	23.73	21.71	32.65	21.93
	MAPE	19.80	40.34	14.70	18.97
	NRMSE	0.11	0.10	0.14	0.10
	TP/P	–	54/127	212/296	1125/1327
	FP/N	–	145/1623	145/1623	156/423
18	RMSE	26.61	30.58	38.80	24.26
	MAE	18.98	21.89	29.50	17.41
	MAPE	14.91	35.85	14.34	14.52
	NRMSE	0.12	0.14	0.18	0.11
	TP/P	–	19/83	280/538	3483/3751
	FP/N	–	191/4289	191/4289	320/621
19	RMSE	34.40	33.32	43.63	31.08
	MAE	24.54	24.83	31.90	22.27
	MAPE	18.18	41.34	14.39	18.34
	NRMSE	0.10	0.09	0.12	0.09
	TP/P	–	90/239	1189/1686	4544/5495
	FP/N	–	704/7181	704/7181	631/1925
20	RMSE	29.82	32.53	44.15	27.06
	MAE	19.90	18.84	32.72	18.20
	MAPE	16.69	33.25	14.76	15.92
	NRMSE	0.10	0.11	0.15	0.09
	TP/P	–	175/420	790/930	5874/6730
	FP/N	–	398/7660	398/7660	379/1350
21	RMSE	25.93	15.47	40.56	23.50
	MAE	19.62	11.37	33.45	18.04
	MAPE	15.24	19.14	15.10	15.02
	NRMSE	0.08	0.05	0.12	0.07
	TP/P	–	241/375	543/909	5449/5993
	FP/N	–	500/6902	500/6902	499/1284
22	RMSE	20.60	15.84	30.61	19.23
	MAE	15.36	11.26	25.04	14.36
	MAPE	12.13	19.11	11.88	11.91
	NRMSE	0.08	0.06	0.11	0.07
	TP/P	–	168/249	406/812	6465/6839
	FP/N	–	347/7651	347/7651	487/1061

23	RMSE	22.78	27.90	29.73	21.15
	MAE	16.13	21.40	20.85	15.16
	MAPE	12.72	34.41	9.25	12.91
	NRMSE	0.07	0.09	0.10	0.07
	TP/P	–	6/141	1059/1265	6616/6938
	FP/N	–	50/8203	50/8203	339/1406
24	RMSE	22.62	18.35	37.63	21.76
	MAE	16.16	13.75	28.41	15.65
	MAPE	14.61	23.22	13.88	14.05
	NRMSE	0.10	0.08	0.16	0.09
	TP/P	–	208/400	217/320	5222/5773
	FP/N	–	390/6093	390/6093	295/720
25	RMSE	25.77	19.75	21.36	26.91
	MAE	20.78	16.05	15.98	22.02
	MAPE	18.82	25.12	7.63	20.25
	NRMSE	0.10	0.07	0.08	0.10
	TP/P	–	370/425	752/871	3646/4968
	FP/N	–	964/5839	964/5839	174/1296
26	RMSE	22.82	23.46	32.86	20.50
	MAE	16.75	17.02	25.45	15.19
	MAPE	13.01	27.66	11.99	12.81
	NRMSE	0.09	0.09	0.12	0.08
	TP/P	–	53/168	798/1158	6112/6476
	FP/N	–	179/7634	179/7634	475/1326
27	RMSE	29.07	27.16	49.46	25.69
	MAE	21.62	20.51	39.68	19.52
	MAPE	18.54	36.00	17.77	17.22
	NRMSE	0.09	0.09	0.16	0.08
	TP/P	–	148/441	378/659	5007/5448
	FP/N	–	314/6107	314/6107	573/1100
28	RMSE	20.78	18.62	28.19	18.80
	MAE	15.09	13.88	21.23	13.76
	MAPE	11.40	23.95	9.73	11.28
	NRMSE	0.07	0.06	0.10	0.06
	TP/P	–	170/268	1160/1510	6259/6697
	FP/N	–	255/8207	255/8207	448/1778
29	RMSE	30.41	25.08	41.39	27.98
	MAE	22.79	17.51	30.54	21.53
	MAPE	17.69	32.80	14.68	17.41
	NRMSE	0.13	0.11	0.18	0.12
	TP/P	–	77/153	332/526	2278/2597
	FP/N	–	280/3123	280/3123	267/679
30	RMSE	17.75	13.16	18.00	17.92
	MAE	13.52	8.76	13.88	13.70
	MAPE	10.83	14.87	6.59	11.66
	NRMSE	0.06	0.05	0.06	0.06
	TP/P	–	270/364	1355/1607	5375/6374
	FP/N	–	663/7981	663/7981	346/1971
31	RMSE	21.47	19.01	26.67	20.85
	MAE	15.19	11.85	19.52	14.89
	MAPE	13.40	19.25	9.27	13.43
	NRMSE	0.08	0.07	0.10	0.08
	TP/P	–	312/617	710/915	5767/6472
	FP/N	–	524/7387	524/7387	509/1532
32	RMSE	20.79	17.83	24.13	20.00
	MAE	15.09	12.92	17.62	14.56
	MAPE	11.73	21.24	8.06	12.33
	NRMSE	0.07	0.06	0.08	0.06
	TP/P	–	101/161	1132/1354	5198/5767
	FP/N	–	352/7121	352/7121	280/1515
33	RMSE	40.06	29.44	54.45	38.60
	MAE	30.82	14.47	38.93	30.91
	MAPE	25.79	25.22	17.92	26.76
	NRMSE	0.13	0.10	0.18	0.13
	TP/P	–	325/369	432/685	2971/5769
	FP/N	–	2451/6454	2451/6454	259/1054

34	RMSE<br>MAE<br>MAPE<br>NRMSE<br>TP/P<br>FP/N	19.89<br>13.02<br>11.00<br>0.07<br>–<br>–	16.35<br>11.25<br>18.46<br>0.06<br>220/332<br>306/5996	30.78<br>21.01<br>9.94<br>0.11<br>474/613<br>306/5996	18.46<br>12.22<br>10.66<br>0.06<br>4942/5383<br>247/945
35	RMSE<br>MAE<br>MAPE<br>NRMSE<br>TP/P<br>FP/N	28.79<br>22.04<br>18.45<br>0.11<br>–<br>–	39.27<br>30.88<br>48.70<br>0.15<br>23/144<br>551/7595	32.78<br>24.39<br>12.01<br>0.13<br>524/733<br>551/7595	28.07<br>21.61<br>18.51<br>0.11<br>5843/6862<br>328/877
36	RMSE<br>MAE<br>MAPE<br>NRMSE<br>TP/P<br>FP/N	24.26<br>18.35<br>15.71<br>0.08<br>–<br>–	17.18<br>9.80<br>16.56<br>0.06<br>196/301<br>1051/7240	27.52<br>20.73<br>9.87<br>0.10<br>651/883<br>1051/7240	24.06<br>18.43<br>16.48<br>0.08<br>5114/6357<br>336/1184
37	RMSE<br>MAE<br>MAPE<br>NRMSE<br>TP/P<br>FP/N	28.05<br>20.52<br>17.19<br>0.09<br>–<br>–	23.25<br>15.09<br>26.33<br>0.08<br>210/384<br>637/6650	40.86<br>27.25<br>12.20<br>0.13<br>575/805<br>637/6650	26.10<br>19.96<br>17.27<br>0.09<br>4903/5845<br>393/1189
38	RMSE<br>MAE<br>MAPE<br>NRMSE<br>TP/P<br>FP/N	25.82<br>19.21<br>16.86<br>0.11<br>–<br>–	17.31<br>12.21<br>20.39<br>0.07<br>447/663<br>739/5639	39.28<br>30.54<br>14.63<br>0.16<br>254/459<br>739/5639	25.21<br>19.10<br>16.61<br>0.11<br>4379/5180<br>419/1122
39	RMSE<br>MAE<br>MAPE<br>NRMSE<br>TP/P<br>FP/N	33.86<br>24.92<br>18.86<br>0.12<br>–<br>–	46.38<br>29.63<br>52.44<br>0.16<br>49/87<br>533/7417	33.57<br>23.18<br>10.79<br>0.12<br>1283/1512<br>533/7417	33.72<br>25.30<br>20.44<br>0.12<br>4531/5905<br>253/1599
40	RMSE<br>MAE<br>MAPE<br>NRMSE<br>TP/P<br>FP/N	22.13<br>15.56<br>9.96<br>0.07<br>–<br>–	15.54<br>12.12<br>18.03<br>0.05<br>11/35<br>25/6143	32.07<br>24.92<br>10.94<br>0.10<br>1221/1601<br>25/6143	17.37<br>12.29<br>9.55<br>0.05<br>4297/4542<br>404/1636
41	RMSE<br>MAE<br>MAPE<br>NRMSE<br>TP/P<br>FP/N	23.03<br>16.20<br>13.28<br>0.06<br>–<br>–	14.23<br>8.85<br>14.96<br>0.04<br>346/485<br>691/6571	28.48<br>22.31<br>9.14<br>0.08<br>865/1027<br>691/6571	22.52<br>15.71<br>13.90<br>0.06<br>4667/5544<br>300/1512
42	RMSE<br>MAE<br>MAPE<br>NRMSE<br>TP/P<br>FP/N	25.92<br>19.62<br>15.90<br>0.11<br>–<br>–	24.24<br>17.18<br>27.41<br>0.10<br>42/100<br>250/4021	28.56<br>20.64<br>10.00<br>0.12<br>526/673<br>250/4021	25.40<br>19.49<br>16.74<br>0.11<br>2914/3348<br>205/773
43	RMSE<br>MAE<br>MAPE<br>NRMSE<br>TP/P<br>FP/N	26.56<br>19.70<br>14.36<br>0.08<br>–<br>–	14.71<br>10.73<br>17.60<br>0.04<br>245/329<br>471/5950	36.80<br>28.72<br>12.23<br>0.11<br>1043/1469<br>471/5950	23.00<br>17.41<br>14.83<br>0.07<br>3896/4481<br>510/1798
44	RMSE<br>MAE<br>MAPE<br>NRMSE<br>TP/P<br>FP/N	23.63<br>17.64<br>14.08<br>0.07<br>–<br>–	19.96<br>10.72<br>18.78<br>0.06<br>391/455<br>1537/7600	25.03<br>18.38<br>8.15<br>0.07<br>2036/2332<br>1537/7600	23.29<br>17.90<br>16.30<br>0.06<br>3504/5268<br>356/2787

45	RMSE	27.23	17.46	40.19	25.94
	MAE	18.99	12.99	26.98	18.69
	MAPE	16.49	22.57	12.56	16.14
	NRMSE	0.08	0.05	0.12	0.08
	TP/P	–	565/788	479/777	4024/4880
	FP/N	–	755/5657	755/5657	517/1565
46	RMSE	19.92	26.64	26.05	18.00
	MAE	14.12	19.21	19.73	12.68
	MAPE	10.18	31.03	9.12	10.26
	NRMSE	0.07	0.10	0.09	0.07
	TP/P	–	15/39	912/1106	4072/4448
	FP/N	–	121/5554	121/5554	218/1145
47	RMSE	18.85	10.45	23.87	17.74
	MAE	13.36	7.32	18.55	12.38
	MAPE	10.12	12.06	8.48	10.43
	NRMSE	0.07	0.04	0.08	0.06
	TP/P	–	91/146	1049/1286	5580/5904
	FP/N	–	191/7190	191/7190	292/1432
48	RMSE	30.85	23.72	33.80	30.34
	MAE	23.07	15.84	25.59	22.87
	MAPE	18.17	29.80	11.11	19.61
	NRMSE	0.09	0.07	0.09	0.08
	TP/P	–	404/550	1688/2006	4059/5391
	FP/N	–	898/7397	898/7397	452/2556
49	RMSE	45.38	34.10	57.56	36.71
	MAE	33.03	21.79	43.03	27.74
	MAPE	21.50	41.01	16.11	23.12
	NRMSE	0.13	0.09	0.16	0.10
	TP/P	–	151/266	1620/1843	2040/2915
	FP/N	–	511/4758	511/4758	325/2109
50	RMSE	37.82	25.13	50.18	34.12
	MAE	27.22	16.15	37.11	25.16
	MAPE	20.24	27.02	16.94	20.65
	NRMSE	0.12	0.08	0.16	0.11
	TP/P	–	350/472	1006/1576	3968/5020
	FP/N	–	783/6596	783/6596	675/2048
51	RMSE	24.62	20.39	30.07	24.23
	MAE	17.61	14.30	21.87	17.42
	MAPE	15.70	24.60	10.18	15.40
	NRMSE	0.07	0.06	0.09	0.07
	TP/P	–	490/791	737/925	5728/6645
	FP/N	–	675/7570	675/7570	489/1716
52	RMSE	23.37	22.76	21.44	23.93
	MAE	17.36	14.46	16.40	17.75
	MAPE	13.31	25.36	7.31	14.61
	NRMSE	0.08	0.08	0.07	0.08
	TP/P	–	67/136	964/1044	3053/3542
	FP/N	–	151/4586	151/4586	149/1180
53	RMSE	32.16	22.22	40.90	27.97
	MAE	22.08	15.12	28.89	19.44
	MAPE	15.08	24.34	12.67	15.74
	NRMSE	0.09	0.07	0.12	0.08
	TP/P	–	74/141	1105/1485	3002/3462
	FP/N	–	290/4947	290/4947	437/1626
54	RMSE	20.10	13.44	22.81	19.71
	MAE	14.49	8.50	16.35	14.44
	MAPE	11.50	14.95	7.11	12.48
	NRMSE	0.06	0.04	0.06	0.05
	TP/P	–	323/471	1509/1667	5130/5774
	FP/N	–	328/7441	328/7441	306/2138
55	RMSE	23.98	34.74	26.86	22.54
	MAE	16.94	25.96	19.94	15.62
	MAPE	11.80	45.32	8.69	12.53
	NRMSE	0.07	0.10	0.07	0.06
	TP/P	–	22/84	1837/2209	5080/5583
	FP/N	–	221/7792	221/7792	432/2293

56	RMSE	24.52	33.03	24.17	24.55
	MAE	18.51	23.81	18.50	18.45
	MAPE	13.30	39.52	8.34	15.05
	NRMSE	0.07	0.10	0.07	0.07
	TP/P	–	23/60	1875/2164	4600/5248
	FP/N	–	161/7412	161/7412	325/2224
57	RMSE	22.43	16.09	28.18	19.52
	MAE	15.59	12.14	21.08	13.25
	MAPE	10.36	19.75	9.41	10.50
	NRMSE	0.07	0.05	0.09	0.06
	TP/P	–	70/145	1662/2164	4575/4867
	FP/N	–	127/7031	127/7031	575/2309
58	RMSE	32.47	24.44	34.40	31.99
	MAE	23.35	13.02	24.03	23.54
	MAPE	17.19	21.73	10.80	19.53
	NRMSE	0.10	0.08	0.11	0.10
	TP/P	–	160/220	1665/1963	3723/4932
	FP/N	–	666/6895	666/6895	347/2183
59	RMSE	20.83	8.67	21.78	20.61
	MAE	15.15	6.67	16.10	14.93
	MAPE	10.68	11.30	6.91	12.35
	NRMSE	0.06	0.03	0.06	0.06
	TP/P	–	123/133	2140/2403	4631/5372
	FP/N	–	409/7775	409/7775	273/2536
60	RMSE	35.06	24.20	45.94	29.64
	MAE	24.31	17.18	32.79	21.04
	MAPE	17.24	29.18	13.92	17.54
	NRMSE	0.10	0.07	0.13	0.08
	TP/P	–	275/549	1820/2429	4357/5103
	FP/N	–	569/7532	569/7532	871/2978
61	RMSE	22.95	37.74	24.94	21.49
	MAE	16.97	27.45	19.26	15.70
	MAPE	11.66	53.99	8.95	11.82
	NRMSE	0.08	0.13	0.09	0.08
	TP/P	–	42/103	1472/1841	3737/4151
	FP/N	–	80/5992	80/5992	430/1944
62	RMSE	32.84	33.20	31.01	34.05
	MAE	25.04	22.73	22.92	26.60
	MAPE	17.23	36.45	10.22	21.53
	NRMSE	0.10	0.10	0.09	0.10
	TP/P	–	61/129	2606/2871	2903/4098
	FP/N	–	426/6969	426/6969	325/3000
63	RMSE	31.66	36.66	34.67	29.37
	MAE	23.70	24.79	26.63	21.83
	MAPE	16.02	41.59	11.20	17.81
	NRMSE	0.09	0.10	0.10	0.08
	TP/P	–	88/205	2267/2702	3700/4355
	FP/N	–	191/7057	191/7057	551/2907
64	RMSE	27.82	23.08	32.22	25.44
	MAE	20.04	14.11	23.32	18.53
	MAPE	13.59	23.16	9.92	15.23
	NRMSE	0.08	0.06	0.09	0.07
	TP/P	–	57/100	2195/2598	4391/5230
	FP/N	–	433/7828	433/7828	443/2698
65	RMSE	27.49	17.86	33.11	25.36
	MAE	18.92	13.17	23.26	17.51
	MAPE	13.54	23.30	10.20	14.21
	NRMSE	0.08	0.05	0.09	0.07
	TP/P	–	330/421	1914/2299	4734/5345
	FP/N	–	364/7644	364/7644	469/2720
66	RMSE	20.15	15.40	24.96	17.55
	MAE	14.59	11.59	18.68	12.73
	MAPE	9.72	19.22	8.39	10.10
	NRMSE	0.06	0.04	0.07	0.05
	TP/P	–	40/111	1611/2005	3996/4246
	FP/N	–	59/6251	59/6251	465/2116

67	RMSE	21.59	31.40	23.68	19.36
	MAE	14.91	24.73	16.91	13.03
	MAPE	9.69	47.02	7.10	10.38
	NRMSE	0.06	0.09	0.07	0.05
	TP/P	–	59/151	2991/3303	3933/4292
	FP/N	–	32/7595	32/7595	404/3454
68	RMSE	32.85	36.88	32.71	32.68
	MAE	25.58	27.26	25.43	25.56
	MAPE	18.38	49.47	10.40	20.97
	NRMSE	0.09	0.10	0.09	0.09
	TP/P	–	29/132	1165/1246	1778/2259
	FP/N	–	62/3505	62/3505	183/1378
69	RMSE	31.13	58.31	30.70	31.09
	MAE	23.34	44.09	23.48	22.99
	MAPE	14.24	74.26	10.08	17.44
	NRMSE	0.09	0.16	0.09	0.09
	TP/P	–	4/41	3074/3442	2885/3709
	FP/N	–	26/7151	26/7151	402/3483
70	RMSE	27.57	15.74	29.95	25.05
	MAE	19.33	11.39	22.23	16.46
	MAPE	10.92	18.11	9.31	12.47
	NRMSE	0.08	0.05	0.09	0.07
	TP/P	–	21/52	3231/3615	3136/3516
	FP/N	–	15/7131	15/7131	415/3667
71	RMSE	24.94	11.65	24.25	26.11
	MAE	18.19	7.45	17.87	19.13
	MAPE	11.97	12.19	7.52	15.62
	NRMSE	0.07	0.03	0.07	0.07
	TP/P	–	180/228	2660/2938	2745/3576
	FP/N	–	492/6514	492/6514	326/3166
72	RMSE	24.78	29.10	24.85	24.70
	MAE	19.09	26.80	18.51	19.56
	MAPE	11.44	47.86	7.74	14.40
	NRMSE	0.07	0.08	0.07	0.07
	TP/P	–	5/20	3561/3850	3747/4561
	FP/N	–	0/8411	0/8411	304/3870
73	RMSE	14.52	No test samples	14.94	13.20
	MAE	10.25	No test samples	10.46	9.62
	MAPE	4.89	No test samples	4.38	6.40
	NRMSE	0.05	No test samples	0.05	0.05
	TP/P	–	No test samples	6115/6316	1824/2146
	FP/N	–	0/8462	0/8462	201/6316
74	RMSE	17.71	20.18	32.73	16.95
	MAE	12.22	15.77	26.35	11.73
	MAPE	9.86	24.18	13.16	9.70
	NRMSE	0.08	0.10	0.16	0.08
	TP/P	–	6/25	137/267	7558/7765
	FP/N	–	97/8032	97/8032	149/292
75	RMSE	17.78	25.85	27.50	16.63
	MAE	12.42	19.03	19.99	11.73
	MAPE	9.90	31.51	9.93	9.69
	NRMSE	0.07	0.11	0.11	0.07
	TP/P	–	14/61	305/511	6037/6228
	FP/N	–	84/6739	84/6739	253/572
76	RMSE	20.77	14.80	37.25	19.03
	MAE	14.80	11.68	28.61	13.79
	MAPE	11.70	17.34	14.19	11.50
	NRMSE	0.11	0.08	0.19	0.10
	TP/P	–	9/25	259/516	6805/7034
	FP/N	–	129/7550	129/7550	273/541
77	RMSE	14.80	14.96	22.22	13.44
	MAE	10.20	11.08	16.57	9.31
	MAPE	7.63	17.00	8.24	7.50
	NRMSE	0.07	0.07	0.11	0.06
	TP/P	–	5/34	707/1024	7117/7329
	FP/N	–	48/8353	48/8353	346/1058

78	RMSE	17.10	21.79	25.48	15.73
	MAE	11.88	17.80	18.86	10.97
	MAPE	9.34	29.72	8.85	9.16
	NRMSE	0.06	0.07	0.09	0.05
	TP/P	–	9/76	533/751	6109/6280
	FP/N	–	21/7031	21/7031	285/827
79	RMSE	20.92	24.44	28.60	19.75
	MAE	14.49	16.68	20.60	13.74
	MAPE	11.55	28.90	9.98	11.45
	NRMSE	0.08	0.09	0.11	0.07
	TP/P	–	50/124	656/877	6962/7446
	FP/N	–	214/8323	214/8323	295/1001
80	RMSE	18.70	18.77	24.56	17.75
	MAE	13.39	14.00	18.35	12.71
	MAPE	9.67	22.14	9.05	9.72
	NRMSE	0.08	0.08	0.11	0.08
	TP/P	–	6/18	615/957	6713/7026
	FP/N	–	40/7983	40/7983	354/975
81	RMSE	18.25	45.62	28.44	16.50
	MAE	13.08	44.13	21.56	12.00
	MAPE	9.69	65.27	9.90	9.63
	NRMSE	0.06	0.15	0.09	0.05
	TP/P	–	0/4	709/892	6928/7119
	FP/N	–	18/8011	18/8011	187/896
82	RMSE	17.00	13.26	20.87	16.42
	MAE	12.45	9.31	16.13	11.97
	MAPE	9.58	13.88	7.88	9.76
	NRMSE	0.08	0.06	0.10	0.08
	TP/P	–	10/28	220/280	1866/1969
	FP/N	–	44/2249	44/2249	78/308
83	RMSE	18.71	40.36	30.13	16.59
	MAE	11.99	31.65	20.77	10.81
	MAPE	8.85	49.76	9.89	8.57
	NRMSE	0.07	0.16	0.12	0.07
	TP/P	–	5/22	601/727	5505/5782
	FP/N	–	47/6509	47/6509	142/749
84	RMSE	20.30	37.50	27.36	19.16
	MAE	14.41	27.19	20.10	13.66
	MAPE	10.65	44.12	9.72	10.65
	NRMSE	0.07	0.12	0.09	0.06
	TP/P	–	3/24	612/903	6910/7266
	FP/N	–	16/8169	16/8169	311/927
85	RMSE	24.46	22.89	34.23	20.96
	MAE	17.81	19.02	26.52	15.36
	MAPE	12.45	30.77	12.35	12.19
	NRMSE	0.08	0.08	0.12	0.07
	TP/P	–	13/95	1117/1661	5603/5941
	FP/N	–	94/7602	94/7602	626/1756
86	RMSE	19.15	14.85	26.60	18.20
	MAE	13.52	9.89	19.76	12.89
	MAPE	10.73	15.33	9.56	10.76
	NRMSE	0.09	0.07	0.12	0.08
	TP/P	–	86/178	582/841	6901/7276
	FP/N	–	192/8117	192/8117	350/1019
87	RMSE	16.47	12.41	23.33	15.06
	MAE	11.32	8.76	16.51	10.48
	MAPE	8.33	13.74	7.56	8.42
	NRMSE	0.05	0.04	0.07	0.04
	TP/P	–	23/50	897/1138	6647/6882
	FP/N	–	69/8020	69/8020	268/1188
88	RMSE	18.19	20.00	23.31	17.23
	MAE	13.00	14.55	17.43	12.29
	MAPE	10.22	23.48	8.16	10.35
	NRMSE	0.07	0.08	0.09	0.07
	TP/P	–	43/100	863/1089	6728/7022
	FP/N	–	139/8111	139/8111	283/1189

89	RMSE	16.69	8.58	27.62	15.72
	MAE	11.61	6.17	17.59	11.26
	MAPE	9.56	9.23	8.62	9.63
	NRMSE	0.09	0.04	0.14	0.08
	TP/P	–	63/99	414/542	7248/7679
	FP/N	–	268/8221	268/8221	164/641
90	RMSE	23.28	18.49	35.78	21.07
	MAE	16.35	13.66	26.92	14.94
	MAPE	12.08	21.68	13.01	11.80
	NRMSE	0.10	0.08	0.15	0.09
	TP/P	–	50/117	597/994	6917/7244
	FP/N	–	221/8238	221/8238	464/1111
91	RMSE	19.61	22.98	24.73	18.36
	MAE	14.14	17.35	19.12	13.11
	MAPE	10.39	29.04	8.75	10.50
	NRMSE	0.06	0.07	0.08	0.06
	TP/P	–	28/80	1041/1387	6476/6903
	FP/N	–	249/8290	249/8290	398/1467
92	RMSE	11.76	No test samples	16.54	10.40
	MAE	8.20	No test samples	11.95	7.37
	MAPE	5.42	No test samples	5.70	5.35
	NRMSE	0.06	No test samples	0.08	0.05
	TP/P	–	No test samples	1308/1526	6611/6865
	FP/N	–	0/8391	0/8391	218/1526
93	RMSE	21.81	33.00	28.77	18.66
	MAE	14.90	29.11	20.25	12.93
	MAPE	10.11	47.34	9.50	10.08
	NRMSE	0.08	0.12	0.11	0.07
	TP/P	–	1/19	763/1046	2839/2976
	FP/N	–	47/4022	47/4022	301/1065
94	RMSE	22.08	45.13	25.09	20.57
	MAE	15.29	33.20	18.09	14.09
	MAPE	10.24	56.96	8.49	10.63
	NRMSE	0.08	0.17	0.09	0.08
	TP/P	–	6/35	1765/2131	5005/5516
	FP/N	–	12/7647	12/7647	394/2166
95	RMSE	19.71	18.82	28.51	16.95
	MAE	13.11	11.59	20.53	11.36
	MAPE	9.22	18.07	9.19	9.12
	NRMSE	0.06	0.05	0.08	0.05
	TP/P	–	43/81	1214/1556	6097/6497
	FP/N	–	143/8053	143/8053	380/1637
96	RMSE	27.87	12.45	35.05	24.67
	MAE	19.43	9.00	25.29	17.27
	MAPE	12.68	15.58	11.39	13.15
	NRMSE	0.09	0.04	0.11	0.08
	TP/P	–	49/70	1706/2153	4992/5523
	FP/N	–	167/7676	167/7676	462/2223
97	RMSE	17.22	37.74	19.37	16.12
	MAE	12.27	36.77	14.20	11.38
	MAPE	8.12	55.70	6.52	8.76
	NRMSE	0.07	0.15	0.08	0.06
	TP/P	–	0/9	2266/2566	5415/5753
	FP/N	–	5/8319	5/8319	309/2575

Supplementary Table S6. Revised 2 – Results for 97 patients at 30 minutes ahead forecasting horizon; RMSE, MAE, MAPE, NRMSE are in mg/dL, TP/P stands for True Positives/Positives while FP/N stands for False Positives/Negatives

2 Figures

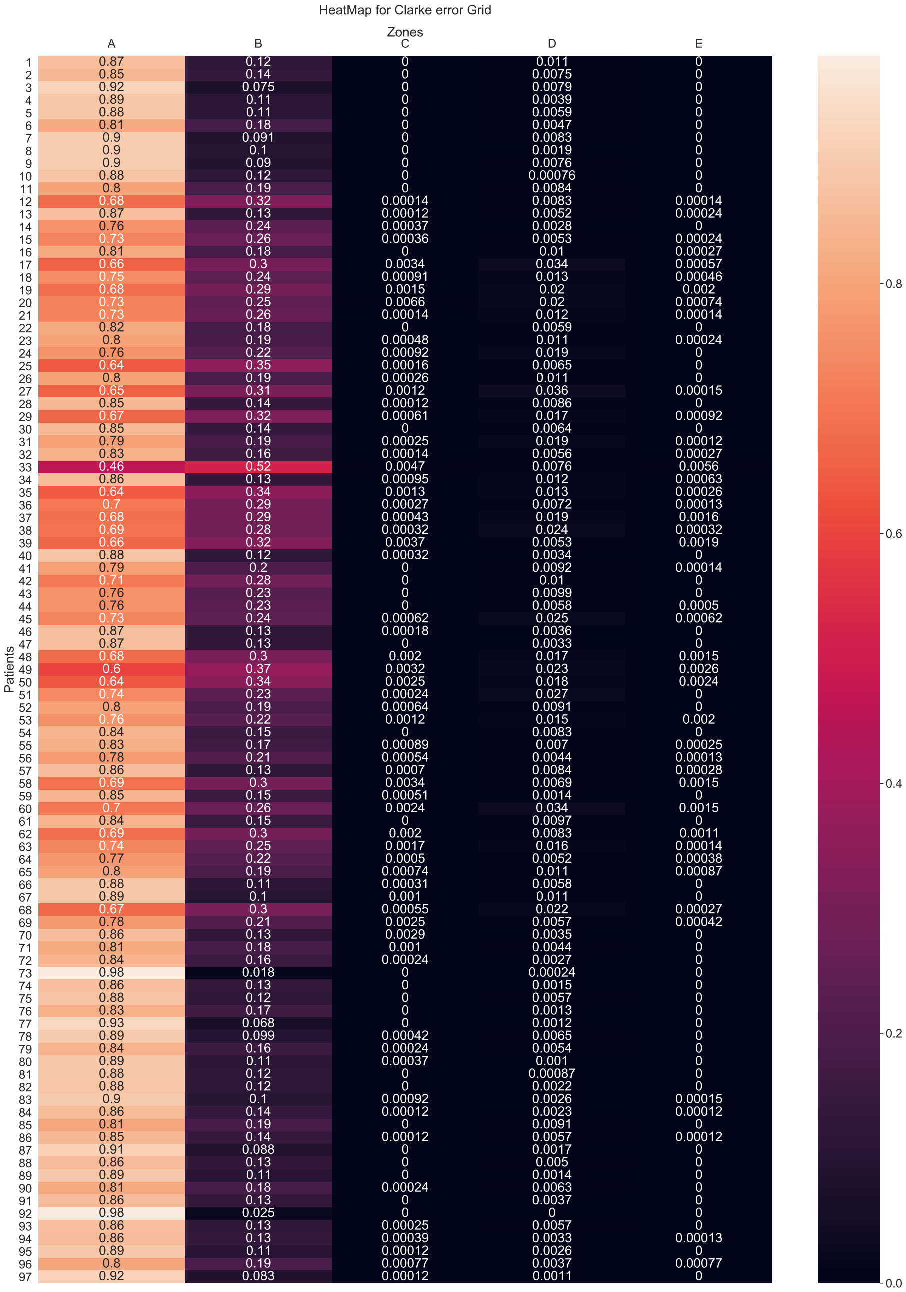

Supplementary Figure S1. HeatMap of Clarke Error Grid

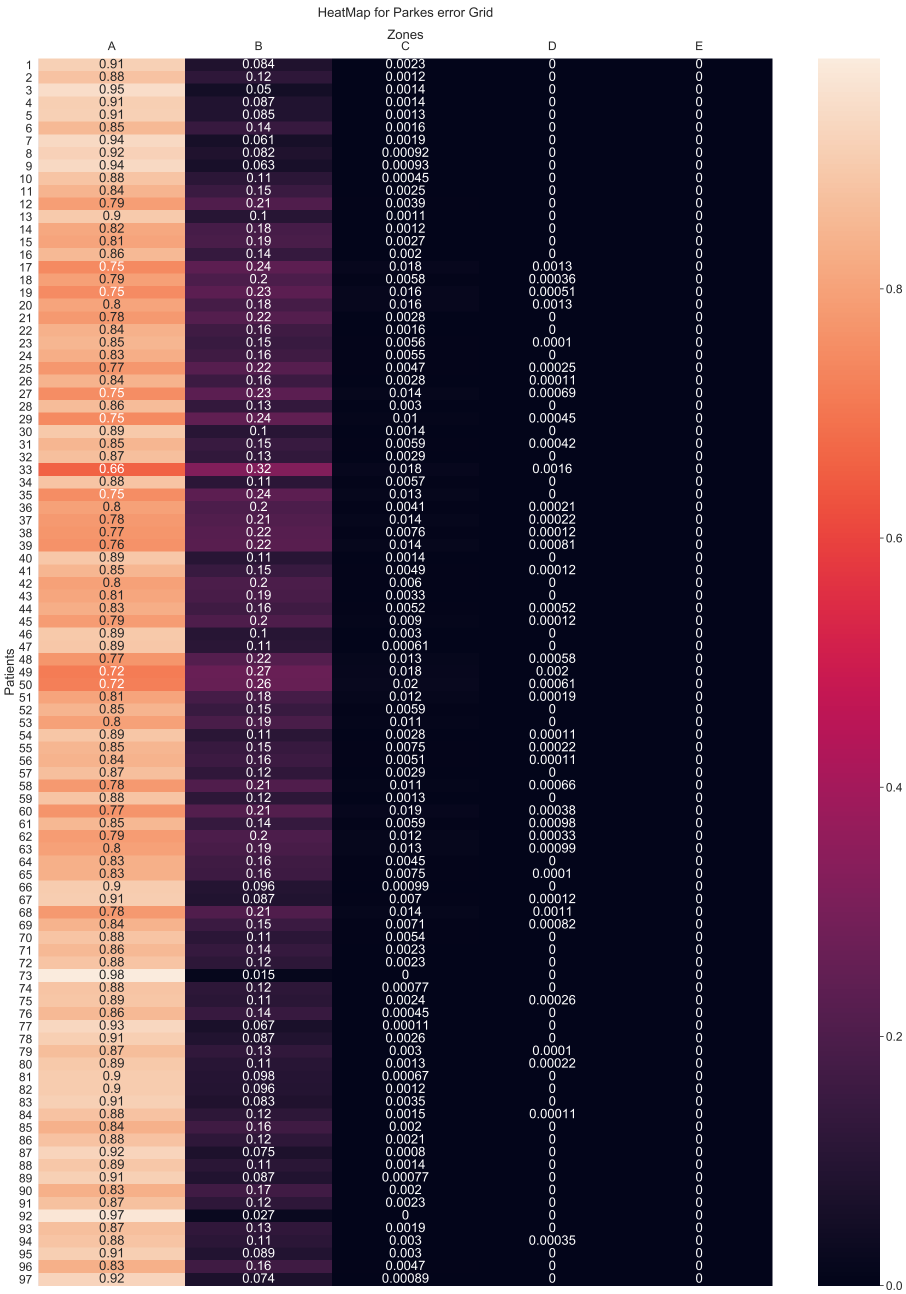

Supplementary Figure S2. HeatMap of Parkes Error Grid
